# Habitat Suitability of Megalopteran Communities in Thailand: Conservation Implications Under Climate Change

**DOI:** 10.3390/insects17100986

**Published:** 2026-09-24

**Authors:** Kanuengnit Wayo, Pranote Yaowapaksopon, Terd Disayathanoowat, Akihiro Nakamura, Xingyue Liu, Watit Khokthong, Kanyakorn Piraonapicha

**Affiliations:** 1 Futuristic Science Research Center, School of Science, Walailak University, Nakhon Si Thammarat 80160, Thailand; kanuengnit.wa@wu.ac.th; 2 Research Center for Theoretical Simulation and Applied Research in Bioscience and Sensing, Walailak University, Nakhon Si Thammarat 80160, Thailand; 3 Environmental Science Program, Faculty of Science, Chiang Mai University, Chiang Mai 50200, Thailand; pranotepium@gmail.com; 4 Department of Biology, Faculty of Science, Chiang Mai University, Chiang Mai 50200, Thailand; terd.dis@cmu.ac.th; 5 Research Center of Deep Technology in Beekeeping and Bee Products for Sustainable Development Goals (SMART BEE SDGs), Chiang Mai University, Chiang Mai 50200, Thailand; 6 Greater Mekong Subregion (GMS) Biodiversity Innovation Research Center (GMS-BIRC), Faculty of Science, Chiang Mai University, Chiang Mai 50200, Thailand; 7 Yunnan Key Laboratory of Forest Ecosystem Stability and Global Change, Xishuangbanna Tropical Botanical Garden, Chinese Academy of Sciences, Menglun, Mengla 666303, China; a.nakamura@xtbg.ac.cn; 8 State Key Laboratory of Animal Biodiversity Conservation and Integrated Pest Management, Institute of Zoology, Chinese Academy of Sciences, Beijing 100101, China; xingyue_liu@yahoo.com; 9 Insect Systematics Research Unit, Queen Sirikit Botanic Garden, The Botanical Garden Organization, Chiang Mai 50180, Thailand

**Keywords:** bioclimatic variables, ecosystem services, land-use change, freshwater insects, Megaloptera, species distribution modeling

## Abstract

Members of the insect order Megaloptera (alderflies, fishflies, dobsonflies) are ecologically important freshwater insects and valuable biological indicators in freshwater biomonitoring programs, yet their spatial patterns and vulnerability to climate change in Southeast Asia remain poorly understood. We compiled 116 occurrence records representing 23 species across seven genera from the Global Biodiversity Information Facility (GBIF) database, published literature, and our field surveys. We examined the distribution of megalopterans across freshwater ecoregions in Thailand, assessed genus-level associations with climatic, topographic, and landscape variables, and projected habitat suitability under current and future climate scenarios. Our results showed significant differences among genera in elevation, proximity to river and canal networks, and climatic factors, including temperature, humidity, and precipitation. Using a range of modeling approaches based on 19 bioclimatic variables from the WorldClim database, we found that habitat suitability for megalopterans is most strongly driven by the mean temperature of the driest quarter (BIO9), precipitation of the wettest month (BIO13), and maximum temperature of the warmest month (BIO5). Our results indicated that approximately 40% of Thailand (209,170 km^2^) currently provides suitable habitat for megalopterans overall. However, future climate scenarios predict substantial habitat loss, with moderate- and high-suitability areas declining sharply by the 2050s and nearly disappearing by the 2090s under high-emission pathways. Our findings underscore the vulnerability of megalopterans in Thailand to climate warming and seasonality, emphasizing the urgent need for freshwater ecosystem conservation and global mitigation efforts to protect tropical aquatic insect diversity.

## Simple Summary

Megalopterans are important freshwater insects and valuable biological indicators in freshwater biomonitoring programs. This study investigated their distribution and potential responses to climate change in Thailand using 116 occurrence records representing 23 species from seven genera. We found that their distributions were strongly related to elevation, distance to rivers and canals, temperature, humidity, and rainfall. Climate models showed that the mean temperature of the driest quarter, precipitation of the wettest month, and maximum temperature of the warmest month were the most important factors determining suitable habitats. At present, about 40% of Thailand is suitable for megalopteran communities. However, suitable habitats are predicted to decline substantially in the future, especially under high-emission climate scenarios, with highly suitable areas becoming very limited by the 2090s. These findings highlight the sensitivity of megalopterans to climate warming and seasonal changes and emphasize the importance of conserving freshwater habitats and reducing climate change impacts.

## 1. Introduction

Climate change is expected to amplify existing hydroclimatic regimes by increasing precipitation variability in highly seasonal regions and intensifying wet seasons in regions with low seasonality [1]. Concurrently, climate change and increasing water abstraction are accelerating the frequency and extent of river drying worldwide [2,3]. These climate-driven hydrological alterations, along with land-use pressures such as riparian deforestation and agricultural expansion, degrade riparian vegetation, impair water quality, reduce macroinvertebrate diversity, and simplify stream channel structure, ultimately diminishing habitat availability and ecosystem functioning [4,5]. Together, these interacting pressures pose compounded risks to freshwater insect communities, which serve as critical indicators of ecosystem health and provide essential services including nutrient cycling, organic matter decomposition, and energy transfer to higher trophic levels [6]. Understanding the environmental factors shaping aquatic insect distributions is therefore crucial for predicting their responses to ongoing environmental change and for identifying suitable habitats under future climatic scenarios.

The insect order Megaloptera (alderflies, fishflies, dobsonflies) represents one of the most ancient lineages within the superorder Neuropterida (Insecta: Holometabola). Fossil evidence indicates that the order originated in the Late Triassic, approximately 210–215 million years ago, with the oldest known larval fossils discovered in Ukraine. The order Megaloptera has exhibited a remarkable evolutionary stasis, with larval morphology remaining largely unchanged since the Mesozoic [7]. According to the latest global checklist, the order comprises approximately 432 described species (approximately 407 extant and 25 extinct) distributed among 47 genera, making it a widespread yet species-poor lineage. Extant megalopterans are classified into two families: Corydalidae, which includes the subfamilies Corydalinae (dobsonflies) and Chauliodinae (fishflies), and Sialidae (alderflies) [8,9,10]. All megalopteran species possess aquatic larvae, whereas the egg, pupae, and adult stages are terrestrial [8]. In Thailand, 22 species of Megaloptera, representing seven genera, have been described [10,11].

Among freshwater insects, Megaloptera constitutes a particularly sensitive taxon for detecting climate-driven habitat changes in tropical Asia. Larvae are specialized predators largely confined to cool, oxygen-rich headwaters, and their gill-bearing morphology reflects strong adaptation to flowing waters with high dissolved oxygen levels [11]. This physiological specialization, combined with their high sensitivity to water quality and well-established bioindicator value [12,13], makes the Megaloptera an ideal focal taxon for assessing how climate change may reshape freshwater insect distributions. Beyond their roles as aquatic predators and bioindicators, adults routinely visit flowers for pollen [14], while larvae contribute to the regulation of aquatic invertebrate communities [12]. Certain species also have cultural and economic importance in East Asia; for instance, larvae of *Protohermes grandis* are traditionally harvested as food in parts of Japan [15]. The global map of freshwater ecoregions, which comprises 426 units across nearly all nonmarine regions (excluding Antarctica, Greenland, and some small islands), provides a key framework for assessing biogeographic and conservation analyses [16]. Given the vulnerability of freshwater species to climate warming [17], identifying the environmental drivers shaping megalopteran distributions is critical for assessing habitat availability and informing freshwater conservation planning.

Species distribution models (SDMs), which integrate species occurrence data with environmental predictors to estimate potential distributions and identify key ecological drivers [18], offer a powerful framework for assessing climate-driven shifts in habitat suitability and guiding conservation planning. Despite the ecological importance and environmental sensitivity of megalopterans, SDMs have rarely been applied to this order (e.g., [19]), in contrast to their widespread use for other aquatic insect groups (e.g., [20,21]). For example, the ‘biomod2’ package [22] provides a comprehensive and flexible platform that encompasses all major stages of species distribution modelling, including data preparation, model calibration with multiple algorithms (e.g., generalized linear and additive models, multivariate adaptive regression splines, random forests, and maximum entropy), model evaluation, and spatial projection [22,23]. Owing to this versatility and robustness, the ‘biomod2’ package has been widely adopted in SDM studies across taxa and regions [24].

Recent regional syntheses highlight significant knowledge gaps in the taxonomy, ecology, and biogeography of Megaloptera in tropical regions. In Brazil, Martins et al. [25] reported 27 megalopteran species across two families and six genera; however, records were unevenly distributed among biomes and hydrographic basins, and immature stages were described for fewer than half of the species. Despite a long history of taxonomic research, large geographic areas remain poorly sampled, underscoring persistent limitations in our understanding of megalopteran distributions, life histories, and conservation status. Comparable data gaps are evident in Southeast Asia, including Thailand, where aquatic insect research has largely focused on diversity and water quality assessments (e.g., [26,27]), while predictive habitat-suitability modelling for Megaloptera, particularly under climate change scenarios, remains scarce.

In this study, we applied habitat suitability modelling to megalopteran communities across Thailand. Specifically, we aimed to: (1) characterize the spatial distribution of megalopteran species across freshwater ecoregions and assess environmental differentiation at the genus level; (2) identify the key bioclimatic drivers shaping habitat suitability for the overall megalopteran community; and (3) project current and future potential distributions under multiple climate change scenarios. By integrating these analyses, our study elucidates climate-driven constraints on megalopteran distributions and identifies areas of potential persistence and vulnerability. These findings can provide a basis for climate-sensitive landscape management and conservation planning in Thailand, with broader implications for freshwater insect conservation across tropical Asia.

## 2. Materials and Methods

### 2.1. Study Area and Occurrence Data

This study was conducted in Thailand, which covers a total area of 515,760 km^2^ [28]. The country is characterized by a diverse range of landscapes, including forests, agricultural lands, and urbanized areas. Forests cover approximately 32.66% of the country’s area (168,431 km^2^), while agricultural lands occupy 54.91% (283,190 km^2^), and urbanized areas account for 6.22% (32,082 km^2^) [29]. Agricultural lands in Thailand consist of rice paddy fields (20.58%), field crops (13.95%), perennial crops (13.76%), orchards (3.29%), and other land uses (3.33%) [29]. The study area was also classified using the Freshwater Ecoregions of the World (FEOW) framework (https://www.feow.org/) (assessed on 12 January 2026), which delineates freshwater ecoregions based on the distribution and composition of freshwater fish species and captures major ecological and evolutionary patterns. In Southeast Asia, including Thailand, ecoregion boundaries were defined using a bottom-up approach that integrated published and unpublished field data with expert assessment, resulting in ecoregions that largely correspond to catchment boundaries [16]. Thailand is divided into 10 freshwater ecoregions: Chao Phraya, Eastern Gulf of Thailand Drainages, Khorat Plateau (Mekong), Kratie–Stung Treng (Mekong), Lower and Middle Salween, Lower Lancang (Mekong), Mae Khlong, Malay Peninsula Eastern Slope, Mekong Delta, and Northern Central Sumatra–Western Malaysia (Figure 1). Among these, the Chao Phraya ecoregion is the largest (167,847 km^2^), whereas the Kratie–Stung Treng (Mekong) ecoregion is the smallest (51 km^2^).

Occurrence data for megalopteran species were compiled from three sources: (1) the Global Biodiversity Information Facility (https://www.gbif.org/) [30] (assessed on 12 January 2026), (2) previous studies [9,10,11,31,32,33,34,35,36], and (3) new data collected through field surveys. Field surveys were conducted during 2025–2026 along streams and rivers in seven provinces of Thailand: Mae Hong Son, Chiang Mai, Phitsanulok, Nakhon Si Thammarat, Nan, Ranong, and Loei. Adult megalopterans were collected using ultraviolet light traps equipped with 10-W fluorescent tubes powered by 12- or 18-V DC batteries. Each trap was positioned near the water’s edge above a collecting jar containing 95% ethanol and operated overnight from 17:30 to 06:00. Specimens were retrieved the following morning, sorted, and preserved in 95% ethanol. Occurrence records from all three sources were then reviewed and integrated into the final dataset used for analysis.

### 2.2. Environmental Characterization of Megalopteran Genera

We characterized the landscape, topographic, and climatic conditions of observed megalopteran occurrences at the genus level across Thailand using eight predictor variables (Table S1). Here, genus was used as a categorical factor, and landscape, topographic, and climatic conditions associated with megalopteran occurrences were the response variables. For landscape variables, the most recent land-use maps of all provinces in Thailand (2019–2022) were obtained from the Land Development Department of Thailand as a polygon shapefile. We generated raster layers to calculate the distance from the nearest patch of forest (D_FOR), agricultural land (D_AGR), urbanized land (D_URB), and river/canal networks (D_RIVER) using the distance() function (‘raster’ package) [37]. For the topographic variable, elevation (ELE) was obtained by downloading the elevation data (SRTM 90 m DEM version 4) from the CIAT-CSI SRTM website (https://srtm.csi.cgiar.org) [38] (assessed on 12 January 2026). Climatic variables, including mean monthly temperature, humidity, and precipitation between 2020 and 2024, were obtained using the get_power() function in the ‘nasapower’ package [39]. These data were then averaged to provide year-round estimates of temperature (AVG_TEMP), humidity (AVG_HUM), and precipitation (AVG_PREC).

Prior to analysis, we excluded one genus (*Ctenochauliodes*) because it had only a single occurrence record. We extracted environmental values for each presence location using the extract() function in the ‘terra’ package. Prior to group comparisons, assumptions of parametric tests were evaluated for each environmental predictor. We assessed normality using Shapiro–Wilk tests, and homogeneity of variances among genera was examined using Levene’s tests implemented in the ‘car’ package [40]. Given the non-normal distributions, heterogeneous variances, and relatively small sample sizes, we evaluated differences among megalopteran genera using Kruskal–Wallis rank-sum tests from the ‘stats’ package [41]. For each predictor, chi-square statistics (χ^2^), degrees of freedom, and raw *p*-values were recorded. To account for multiple testing across predictors, Kruskal–Wallis *p*-values were adjusted using the Benjamini–Hochberg false discovery rate (FDR) procedure. When a Kruskal–Wallis test indicated a significant effect (*p* < 0.05), pairwise comparisons among genera were conducted using Dunn’s post-hoc tests implemented in the ‘FSA’ package [42], with Bonferroni correction applied to adjusted *p*-values. Significant differences among genera were summarized using compact letter displays generated with the ‘rcompanion’ package [43], where different letters indicate statistically significant differences for a given predictor. Mean values (±standard deviation) were calculated for each environmental variable and displayed for each genus.

### 2.3. Bioclimatic Variable Selection for Habitat Suitability Modelling

For the current climate (1970–2000), 19 bioclimatic variables (BIO1 to BIO19) were obtained from the WorldClim database (version 2.1) [44] at approximately 30-arc-second resolution (~1 km) to represent mean annual conditions, seasonality, and extreme environmental factors (e.g., temperature and precipitation). Future climate data were sourced from the BCC-CSM2-MR model under four Shared Socioeconomic Pathways (SSPs): SSP126, SSP245, SSP370, and SSP585, for the periods 2041–2060 (2050s) and 2081–2100 (2090s). The bioclimatic datasets were processed by cropping and masking to the spatial extent of Thailand. To minimize spatial autocorrelation and sample bias, spatial thinning was applied with a distance of 1 km. The occurrence data were processed using the ‘spThin’ package [45], resulting in 61 presence points for model development. To identify and remove highly correlated predictors, the bioclimatic variables were filtered using the Variance Inflation Factor (VIF) method via the ‘usdm’ package [46] on the values extracted at the thinned occurrence points. Variables with a Pearson’s correlation coefficient ≥0.8 were iteratively removed using the vifcor() function. The remaining variables were further filtered using VIF analysis, removing variables until all remaining predictors had a VIF score less than 10 using the vifstep() function. The six final bioclimatic variables used in the modeling process were: mean diurnal range (BIO2); maximum temperature of the warmest month (BIO5); mean temperature of the driest quarter (BIO9); precipitation of the wettest month (BIO13); precipitation of the driest month (BIO14); and precipitation of the warmest quarter (BIO18) (Figures S1 and S2).

### 2.4. Habitat Suitability Modelling

To assess the importance of the final bioclimatic variables influencing the habitat suitability of megalopteran species, we set three permutation runs for each variable using the BIOMOD_Modeling() function (‘biomod2’ package) during the modeling process to estimate variable importance scores. We then calculated the averaged variable importance scores and their standard deviations across the permutation runs for each variable. High values indicate predictors that strongly influence model predictions, whereas values close to zero indicate little or no influence.

Habitat suitability models for Megaloptera were developed using the ‘biomod2’ package. We employed 10 predictive algorithms available in this package: artificial neural networks (ANN), classification tree analysis (CTA), flexible discriminant analysis (FDA), generalized additive models (GAM), generalized boosting method (GBM), generalized linear models (GLM), multivariate adaptive regression splines (MARS), maximum entropy (MAXENT), random forest (RF), and the surface range envelope (SRE/BIOCLIM). Pseudo-absences (PAs) were generated because true absence records are not available for Megaloptera. We used the Surface Range Envelope (SRE) strategy, which identifies pseudo-absences from environmental conditions that fall outside the species’ observed environmental envelope [22]. This method is appropriate when species occurrences span a broad range of environmental space, as is the case for Megaloptera, whose records include both pristine headwater streams and human-modified landscapes. Three pseudo-absence datasets were generated, with the number of pseudo-absences matching the number of presences [47].

Model calibration and validation were carried out using 5-fold cross-validation. For each fold, one subset served as the validation dataset while the remaining four subsets were used for model calibration. All 10 algorithms were run across 15 datasets (3 pseudo-absence sets ×5 folds), using the default settings. Model performance was evaluated using two widely adopted metrics: the Area Under the Receiver Operating Characteristic Curve (AUC) and the True Skill Statistic (TSS). AUC is a threshold-independent indicator of predictive accuracy, where values near 0.5 indicate random performance [48]. TSS is threshold-dependent (Sensitivity + Specificity − 1) and ranges from −1 to +1, with values near zero indicating poor predictive ability [49]. Algorithm-specific performance was summarized using the bm_PlotEvalMean function.

The best-performing algorithm, random forest (RF), was selected based on AUC and TSS (Figure S3), and this model was used for all subsequent projections. The final RF model, calibrated using all available runs and datasets, was projected onto the study region to generate habitat suitability maps for current climate conditions. A clamping mask was applied to identify areas of prediction uncertainty, highlighting regions where environmental conditions fall outside the model’s calibration range [22].

### 2.5. Predicting the Potential Distribution Under Current and Future Climate Scenarios

The selected random forest model was used to project habitat suitability for megalopterans across Thailand under current climate conditions. The resulting output was a continuous probability raster, with values ranging from 0 (unsuitable) to 1 (highly suitable). This model was then applied to project habitat suitability across eight future climate scenarios, consisting of four Shared Socioeconomic Pathways (SSPs: SSP126, SSP245, SSP370, SSP585) and two future time periods (2050s: 2041–2060, and 2090s: 2081–2100).

To categorize the continuous suitability projections into distinct classes, fixed thresholds were applied: unsuitable (<0.25), low suitability (0.25 ≤ probability < 0.50), moderate suitability (0.50 ≤ probability < 0.75), and high suitability (≥0.75). In addition to mapping habitat suitability for the current climate, maps were also generated with overlays for forest and river/canal layers. For each climate scenario, the area (in km^2^) corresponding to each of the four suitability classes was calculated using the ‘terra’ package [50], with the cellSize() function to determine raster cell area and logical masks to filter the suitability categories. The area calculations were then summed across the masked regions to obtain the total area for each suitability class. Additionally, the extent of potential human-modified or non-natural habitats (excluding forests) was quantified, providing insights into areas that may require conservation attention, alongside core forest habitats essential for Megaloptera conservation. All analyses in this study were performed using the R program version 4.4.2 [41].

## 3. Results

### 3.1. Environmental Characterization of Megalopteran Communities

The compiled occurrence dataset (Table 1) comprised 116 records across seven genera in Thailand, representing 22 identified species and one additional *Protohermes* record that could not be resolved to species level (Figure 2). The spatial distribution of megalopteran species across seven of the ten freshwater ecoregions in Thailand varied substantially (Figure 3). The Chao Phraya ecoregion, which is also the largest freshwater ecoregion in Thailand, exhibited the highest diversity with 19 species, dominated by *Neochauliodes punctatolosus* and *Protohermes tonkinensis*. The Khorat Plateau (Mekong) ecoregion recorded five species, predominantly *Indosialis bannaensis* and *Neochauliodes punctatolosus*. The Lower & Middle Salween and Malay Peninsula Eastern Slope ecoregions each harbored only two species. The Lower Lancang (Mekong) ecoregion also displays notable biodiversity, with eight recorded species, including a high frequency of *Indosialis bannaensis* and *Neochauliodes tonkinensis* (Figure 2C,G). The Mae Khlong ecoregion exhibited relatively low species richness, with only three species recorded. The lowest species richness was observed in the Northern Central Sumatra–Western Malaysia ecoregion, which recorded only a single species, *Protohermes sumatrensis*.

Kruskal–Wallis tests revealed significant differences among Thai megalopteran genera for eight environmental predictors (Table S3). Elevation (ELE) showed a significant effect (χ^2^ = 28.43, df = 5, *p_adj_* < 0.001), indicating that genera differed markedly in their elevational distributions. Distance to river or canal networks (D_RIVER) was also significant (χ^2^ = 15.67, df = 5, *p_adj_* = 0.013). Climatic variables exhibited particularly strong effects: average temperature (AVG_TEMP; χ^2^ = 34.90, df = 5, *p_adj_* < 0.001), average humidity (AVG_HUM; χ^2^ = 27.34, df = 5, *p_adj_* < 0.001), and average precipitation (AVG_PREC; χ^2^ = 33.88, df = 5, *p_adj_* < 0.001) all differed significantly among genera, highlighting pronounced climatic niche differentiation. In contrast, landscape variables related to human land use, including distance to forest (D_FOR), agricultural land (D_AGR), and urbanized areas (D_URB), did not show statistically significant differences among genera (*p_adj_* > 0.05).

Significant differences among genera based on Dunn’s post-hoc tests were detected for elevation, temperature, humidity, precipitation, and distance to rivers (Figure 4; Table S4). Elevation (ELE) varied among genera, with *Neochauliodes* (801.65 ± 305.40 m) and *Protohermes* (762.62 ± 360.43 m) forming a higher-elevation group, *Indosialis* (403.28 ± 382.17 m) and *Nevromus* (365.82 ± 227.79 m) forming a lower-elevation group, and *Acanthacorydalis* (498.06 ± 319.97 m) and *Neoneuromus* (751.18 ± 376.88 m) exhibiting intermediate values that did not differ significantly from either group. Distance to rivers or canals (D_RIVER) also differed among genera, with *Nevromus* (7670.46 ± 4934.29 m) occurring significantly closer to rivers than *Neochauliodes* (12,018.93 ± 4402.83 m), while *Acanthacorydalis*, *Indosialis*, *Neoneuromus*, and *Protohermes* showed intermediate and overlapping distances. Annual mean temperature (AVG_TEMP) exhibited multiple overlapping significance groups: *Acanthacorydalis* (23.16 ± 0.23 °C) and *Protohermes* (24.16 ± 1.27 °C) formed one group, *Indosialis* (25.55 ± 1.01 °C) another, and *Nevromus* (25.59 ± 1.43 °C), *Neochauliodes* (24.14 ± 1.03 °C), and *Neoneuromus* (23.77 ± 0.43 °C) occupied intermediate positions. For annual mean humidity (AVG_HUM), *Nevromus* formed a distinct group with significantly higher humidity values (81.37 ± 3.25%) compared with all other genera, which showed similar and overlapping means (approximately 76–78%). A comparable pattern was observed for annual mean precipitation (AVG_PREC), where *Nevromus* (5.87 ± 1.80 mm) differed significantly from *Neochauliodes* (4.00 ± 0.53 mm), *Neoneuromus* (3.78 ± 0.09 mm), and *Protohermes* (4.22 ± 1.05 mm), while *Acanthacorydalis* (4.04 ± 0.21 mm) and *Indosialis* (4.12 ± 0.23 mm) showed intermediate and overlapping values.

### 3.2. Response Curves of Key Bioclimatic Variables Influencing the Distribution of Megaloptera

Among the bioclimatic predictors, mean temperature of the driest quarter (BIO9) emerged as the most influential variable, followed by precipitation of the wettest month (BIO13), maximum temperature of the warmest month (BIO5), precipitation of the warmest quarter (BIO18), mean diurnal temperature range (BIO2) and precipitation of the driest month (BIO14), respectively (Figure S4). Response curves showed nonlinear relationships between megalopteran occurrence probability and the six bioclimatic variables. Occurrence probability was relatively stable across intermediate mean diurnal temperature ranges (BIO2) of approximately 8–12 °C (Figure 5A). In contrast, maximum temperature of the warmest month (BIO5) displayed a clear threshold response, with high occurrence below ~32 °C followed by a sharp decline (Figure 5B). Mean temperature of the driest quarter (BIO9) showed a similar negative trend, with occurrence probability decreasing notably beyond ~20 °C (Figure 5C). Precipitation variables also had strong effects. Precipitation of the wettest month (BIO13) peaked at approximately 200–250 mm and declined thereafter (Figure 5D), while precipitation of the driest month (BIO14) exhibited a unimodal response with peak occurrence at ~30–40 mm (Figure 5E). Precipitation of the warmest quarter (BIO18) was negatively associated with occurrence, with probabilities approaching zero above ~500 mm (Figure 5F).

### 3.3. Habitat Suitability Predictions at Current Climate and Priority Anthropogenic Habitats

Under current climatic conditions (1970–2000 baseline), habitat suitability for the megalopteran community showed clear spatial structuring across Thailand. When considering all habitat types combined, unsuitable areas dominated the landscape, accounting for 307,843.3 km^2^ (59.9% of Thailand). Suitable habitats were more limited, with low-, moderate-, and high-suitability classes covering 15.4% (79,076.1 km^2^), 14.3% (73,530.6 km^2^), and 11.0% (56,563.8 km^2^) of the total area, respectively (Figure 6A; Table 2 and Table S5).

Within anthropogenic, non-forest habitats, unsuitable areas were even more prevalent, encompassing 258,866.9 km^2^ (50.4% of the total area). Suitable anthropogenic areas were limited, with low suitability covering 7.6% (39,148.3 km^2^), moderate suitability 7.0% (35,933.1 km^2^), and high suitability only 2.9% (14,978.2 km^2^) (Figure 6B; Table S5).

### 3.4. Potential Suitable Habitats for Future Climate Scenarios

Projected habitat suitability for the Megaloptera community during the 2050s (2041–2060) indicated substantial declines across all future climate scenarios (SSP126–SSP585) compared with current conditions (Figure 7; Table 2 and Table S5). Under SSP126, unsuitable areas expanded to 362,299.3 km^2^ (70.5% of Thailand), while moderate and high suitability habitats were greatly reduced, together accounting for only 6.1% of the total area, with high suitability declining to 0.2% (1270.1 km^2^). Similar patterns were observed under SSP245, where unsuitable areas increased further to 72.6%, and high suitability remained extremely limited (0.5%). Under more severe emission scenarios, habitat contraction intensified. In SSP370, unsuitable areas covered 71.4% of the landscape, and high suitability declined to 0.2%. The most pronounced reduction occurred under SSP585, where unsuitable habitat reached 73.4%, moderate suitability dropped to 2.2%, and high suitability was nearly eliminated, representing only 0.1% (339.1 km^2^) of the total area.

Projections for the late 21st century (2090s; 2081–2100) indicated a further and more severe contraction of suitable habitats for the megalopteran community under all climate scenarios (Figure 8; Table 2 and Table S5). Under SSP126, unsuitable areas expanded to 380,556.8 km^2^ (74.0%), while moderate suitability declined to 3.9% and high suitability was nearly eliminated (<0.1%). Similar patterns were observed under SSP245, where unsuitable habitats covered 74.9% of the landscape and high suitability remained extremely limited (0.1%). Under higher-emission scenarios, habitat loss intensified markedly. In SSP370, unsuitable areas increased to 76.5%, with moderate suitability reduced to 1.2% and high suitability virtually absent. The most extreme decline occurred under SSP585, where unsuitable habitat dominated 81.3% of the total area, low suitability accounted for the remaining 19.2%, and both moderate and high suitability were almost completely lost (<0.2%).

## 4. Discussion

Our results indicate that megalopteran communities in Thailand are primarily structured by topographic and climatic gradients. Significant differences among genera were observed in elevation, proximity to river and canal networks, and climatic factors, including temperature, humidity, and precipitation. Mean temperature of the driest quarter and precipitation of the wettest month emerged as the most influential bioclimatic drivers of habitat suitability. Future climate projections further suggest substantial habitat contraction, with moderate- and high-suitability areas declining markedly by the 2050s and becoming largely absent by the 2090s under high-emission scenarios.

### 4.1. Environmental Structuring of Megalopteran Genera in Thailand

Our results indicate clear environmental structuring of megalopteran genera across Thailand’s freshwater ecoregions, with species richness highest in the Chao Phraya ecoregion, followed by the Lower Lancang and Khorat Plateau (Mekong) regions. Large river basins with extensive, heterogeneous drainage networks are likely to support higher freshwater biodiversity by providing a broader range of thermal, hydrological, and habitat conditions. Consistent with Heino et al. [51], regional species richness appears to constrain local stream macroinvertebrate richness, while local factors such as stream size, water chemistry, and habitat heterogeneity further influence species presence patterns. Aquatic insect communities are therefore hierarchically structured, with broad-scale climatic and landscape variables defining the regional species pool and local environmental filters shaping community assembly [52]. Moreover, at local scales, substrate composition, organic matter availability, hydraulic conditions, and channel morphology likely interact with spatial processes to shape communities, with seasonal variation affecting the relative importance of these drivers [53,54]. Consequently, ecoregions with lower species richness may reflect more limited freshwater habitat availability, reduced environmental heterogeneity, or lower sampling effort.

Our findings also revealed broad elevational patterns at the genus level across Thailand, with lower-elevation genera (*Indosialis* and *Nevromus*), higher-elevation genera (*Neochauliodes* and *Protohermes*), and intermediate distributions for *Acanthacorydalis* and *Neoneuromus*. This suggests that elevation is an important factor shaping megalopteran communities, although species-level analyses are needed to resolve finer-scale elevational responses and ecological niches. It is consistent with Zhang et al. [55], who examined elevational gradients and seasonal shifts in macroinvertebrate diversity in the upper Jinsha River, southeast of the Qinghai–Tibet Plateau, and found that taxonomic diversity increased with elevation while functional diversity declined due to resource constraints. More broadly, across elevational gradients, species richness and abundance of aquatic invertebrates tend to decline, whereas beta diversity increases due to species turnover [56], reflecting strong spatial structuring of communities and ecosystem functioning [57].

Annual mean temperature, humidity, and precipitation played key roles in shaping the distribution of megalopteran genera. Notably, *Nevromus* was associated with higher humidity and precipitation, indicating a specialization for wetter environments. Given their strong habitat specialization and limited dispersal capacity—Corydalidae are typically associated with cold, high-gradient streams, and Sialidae with fine-sediment habitats—megalopterans are particularly sensitive to freshwater habitat alteration [58]. As aquatic larvae, megalopterans function as benthic predators and contribute to energy flow and material cycling in stream ecosystems, although their ecological roles remain underdocumented in many regions [12]. Seasonal environmental variation can further influence functional composition, reinforcing the importance of considering both spatial and temporal dynamics in freshwater biodiversity conservation and management [54,59].

Finally, distance to river or canal networks also differentiated genera, with *Nevromus* occurring significantly closer to rivers than *Neochauliodes*. These differences likely reflect genus-specific preferences related to stream size, flow permanence, or channel structure. However, broad-scale land-cover variables may not fully capture local aquatic habitat conditions, as streams and riparian zones often represent only a small proportion of the surrounding landscape. Thus, areas with relatively low forest cover may still support diverse megalopteran communities if local streams and riparian habitats remain intact. In agreement with Heino et al. [60], both spatial factors—such as proximity to watercourses and geographic context—and local environmental conditions, including stream size, pH, and nutrient availability, jointly shape macroinvertebrate taxonomic and functional structure. Spatial constraints tend to be more influential at broader, across-ecoregion scales, whereas local environmental variables more strongly affect functional traits, emphasizing the importance of multiple ecological filters when interpreting community distributions. Therefore, our habitat suitability estimates should be interpreted as landscape-scale patterns rather than direct assessments of individual stream conditions.

### 4.2. Contribution of Key Bioclimatic Variables

The distribution of megalopteran communities in Thailand was primarily influenced by three key bioclimatic variables: maximum temperature of the warmest month (BIO5), mean temperature of the driest quarter (BIO9), and precipitation of the wettest month (BIO13). These were identified as the most influential factors (variable importance: 10%, 13%, and 11%, respectively), confirming that temperature regimes play a critical role in niche partitioning among Thai megalopterans. Among these, the mean temperature of the driest quarter emerged as the strongest predictor, with a marked decline in habitat suitability beyond approximately 20 °C, indicating that thermal conditions during the dry season critically limit population persistence. The maximum temperature of the warmest month also exhibited a clear threshold response, with high predicted occurrence below ~32 °C and a sharp decline at higher temperatures. This pattern aligns with the strong influence of water temperature on aquatic insect physiology, including growth, metabolism, emergence, fecundity, behavior, and survival [61]. Upper thermal limits identified for macroinvertebrate families, including thermally sensitive groups such as Corydalidae, further emphasize vulnerability to elevated temperatures, even when some acclimation capacity exists [61].

Precipitation of the wettest month was another important driver of habitat suitability, with genera such as *Nevromus* strongly associated with higher rainfall, reflecting specialization for moist, high-flow environments. Wang et al. [62] showed that *Nevromus* is distributed across regions with strong climatic seasonality and large thermal amplitudes and exhibits signatures of positive selection, suggesting evolutionary adaptation to environments where wet-season hydrological stability is critical. Although the genus spans a broad climatic range, our results indicate that, at regional scales in Thailand, *Nevromus* occurrence is constrained by precipitation, likely because high rainfall promotes stream persistence and suitable larval habitats. Moreover, adequate precipitation supports stream permanence, discharge stability, and the maintenance of suitable larval habitats, which are essential for survival and development of aquatic insects [12,59]. Seasonal variability in stream flow can strongly structure aquatic communities, as demonstrated in systems characterized by predictable floods and droughts, where hydrological seasonality influences taxonomic richness and local community composition [63]. Consequently, shifts in precipitation regimes are likely to disproportionately affect species’ persistence and community structure, even when some functional traits show relative resilience.

### 4.3. Changes in Potential Habitat Area Under Current and Future Climate Scenarios

Overall, our projections indicate a substantial climate-driven contraction of suitable habitats for megalopterans in Thailand, with losses becoming more pronounced under future warming scenarios. However, because occurrence records were pooled across species and were unevenly represented among taxa, these projected changes should be interpreted as broad-scale climatic suitability associated with the pooled records rather than as equivalent responses across all megalopteran species. This contraction is consistent with expectations that warming and altered precipitation regimes degrade freshwater habitats through increased thermal stress, reduced base flows, and elevated evaporative demand [61,63]. Tropical insects are especially vulnerable to such changes because they operate close to their thermal optima and have relatively narrow thermal tolerance ranges [64]. Although tropical aquatic macroinvertebrates can tolerate short-term hypoxia, moderately low dissolved oxygen levels may cause strong sublethal effects, particularly reduced insect emergence. These effects could impair long-term population persistence under warming and low-flow conditions [65]. Moreover, bioclimatic envelope models project that more than half of European stream macroinvertebrate species may lose up to 40% of their climatically suitable area, with cold-adapted and endemic taxa particularly affected [66]. In tropical regions such as Thailand, where opportunities for poleward or elevational range shifts are limited, climate-driven habitat loss is likely to be even more severe. When combined with ongoing land-use pressures that significantly reduce local biodiversity [67], these projections highlight the high risk of continued habitat loss for megalopterans and emphasize the urgent need for climate-informed freshwater conservation [68,69]. Future studies using larger and more evenly distributed occurrence datasets should develop species- or genus-specific models to better assess taxon-specific responses to climate change.

## 5. Conclusions

Megalopterans have limited dispersal capacity due to their obligately aquatic larvae and weakly flying adults, resulting in slow range expansion and distributions primarily shaped by long-term climatic and tectonic processes [70]. Although climate warming may promote shifts toward higher elevations in adjacent regions, this limited dispersal ability constrains their capacity to track suitable conditions, increasing vulnerability to habitat loss and fragmentation, particularly in tropical landscapes. Our results demonstrate that temperature and precipitation are key determinants of habitat suitability and that moderate- and high-suitability habitats are projected to decline sharply under future climate scenarios. Consequently, freshwater ecosystems that provide cool, humid, and hydrologically stable conditions, such as headwater streams, intact riparian zones, and well-connected river networks, are likely to serve as critical refugia and should be prioritized in conservation planning. Fine-scale habitat studies further indicate that megalopteran larvae exhibit narrow preferences for flow velocity and water depth, underscoring the importance of maintaining environmental flows under increasing hydrological variability [71]. The rapid loss of high-suitability habitats under high-emission scenarios highlights the need for climate mitigation. It supports the use of freshwater ecoregions and Megaloptera as indicator taxa for monitoring climate impacts and guiding adaptive freshwater conservation.

## Figures and Tables

**Figure 1:**
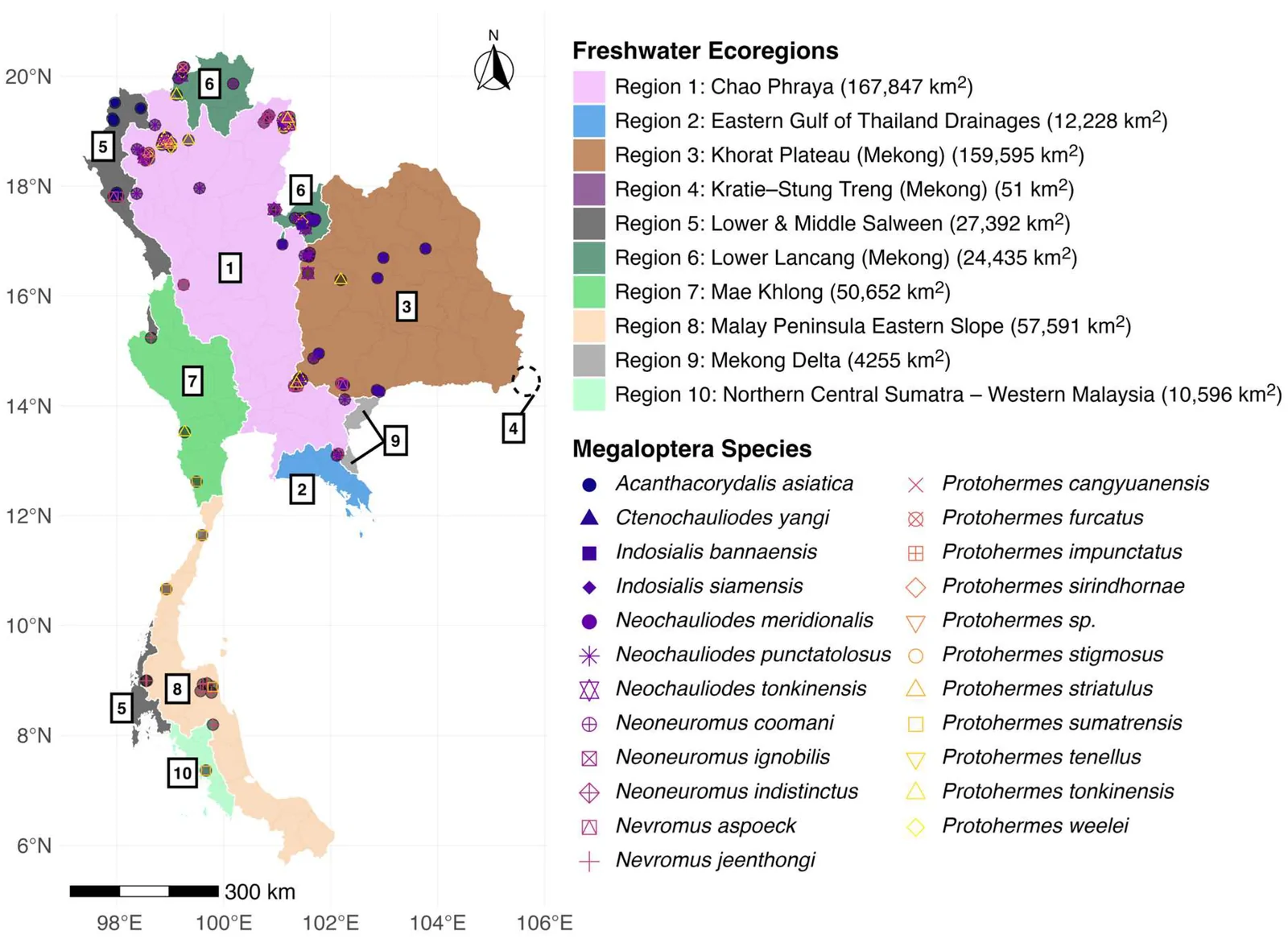
Study area and the 116 geographical distribution records of megalopteran occurrences, representing 22 identified species and one additional *Protohermes* record unresolved to species level, across 10 freshwater ecoregions in Thailand. Each point represents an occurrence record, with different colors and symbols indicating different species.

**Figure 2:**
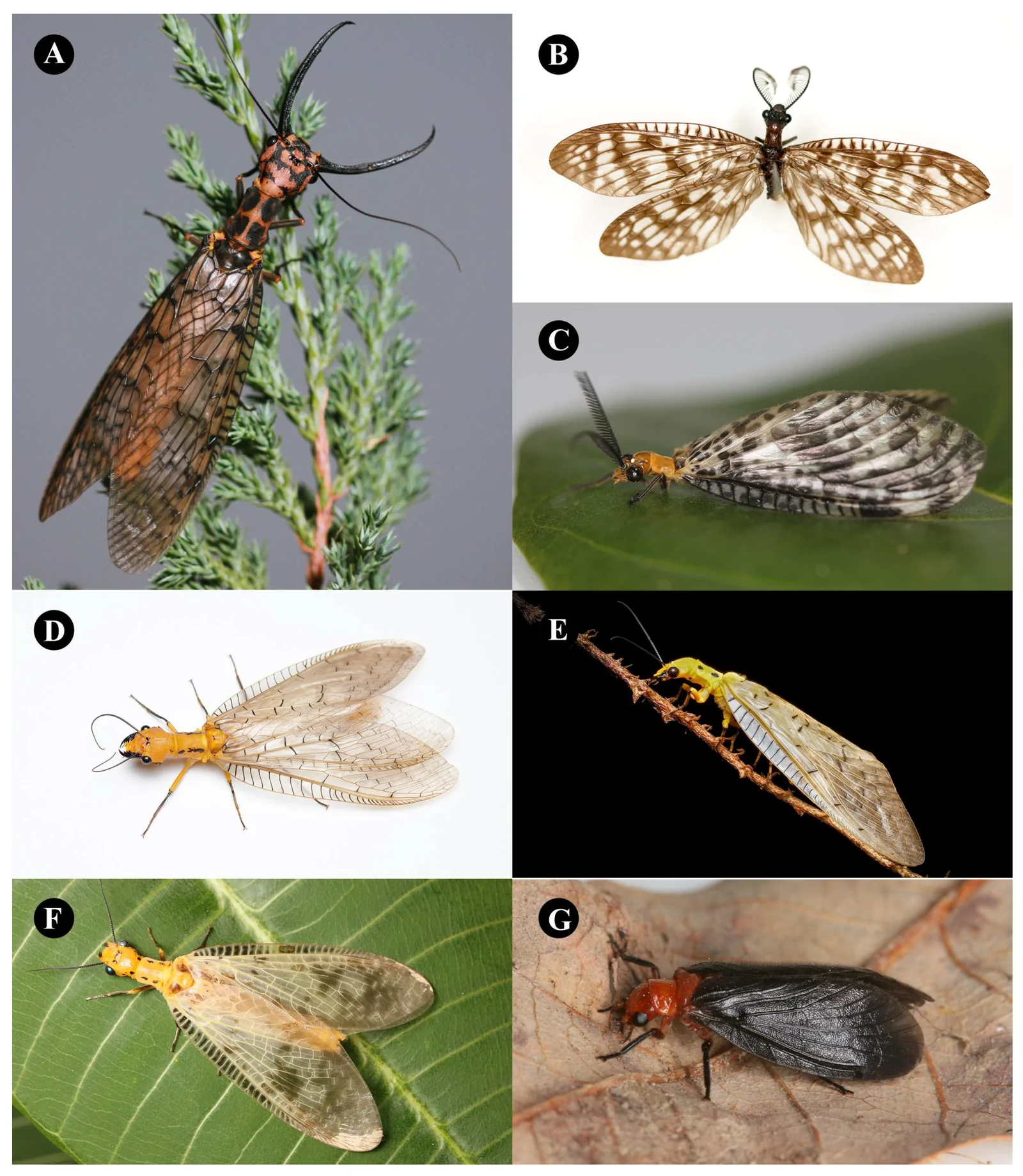
Adults of representative megalopteran species from seven genera in Thailand: *Acanthacorydalis asiatica* (**A**); *Ctenochauliodes yangi* (**B**); *Neochauliodes punctatolosus* (**C**); *Neoneuromus indistinctus* (**D**) (Photo: Wuttipon Pathomwattananurak); *Nevromus jeenthongi* (**E**); *Protohermes weelei* (**F**) and *Indosialis bannaensis* (**G**).

**Figure 3:**
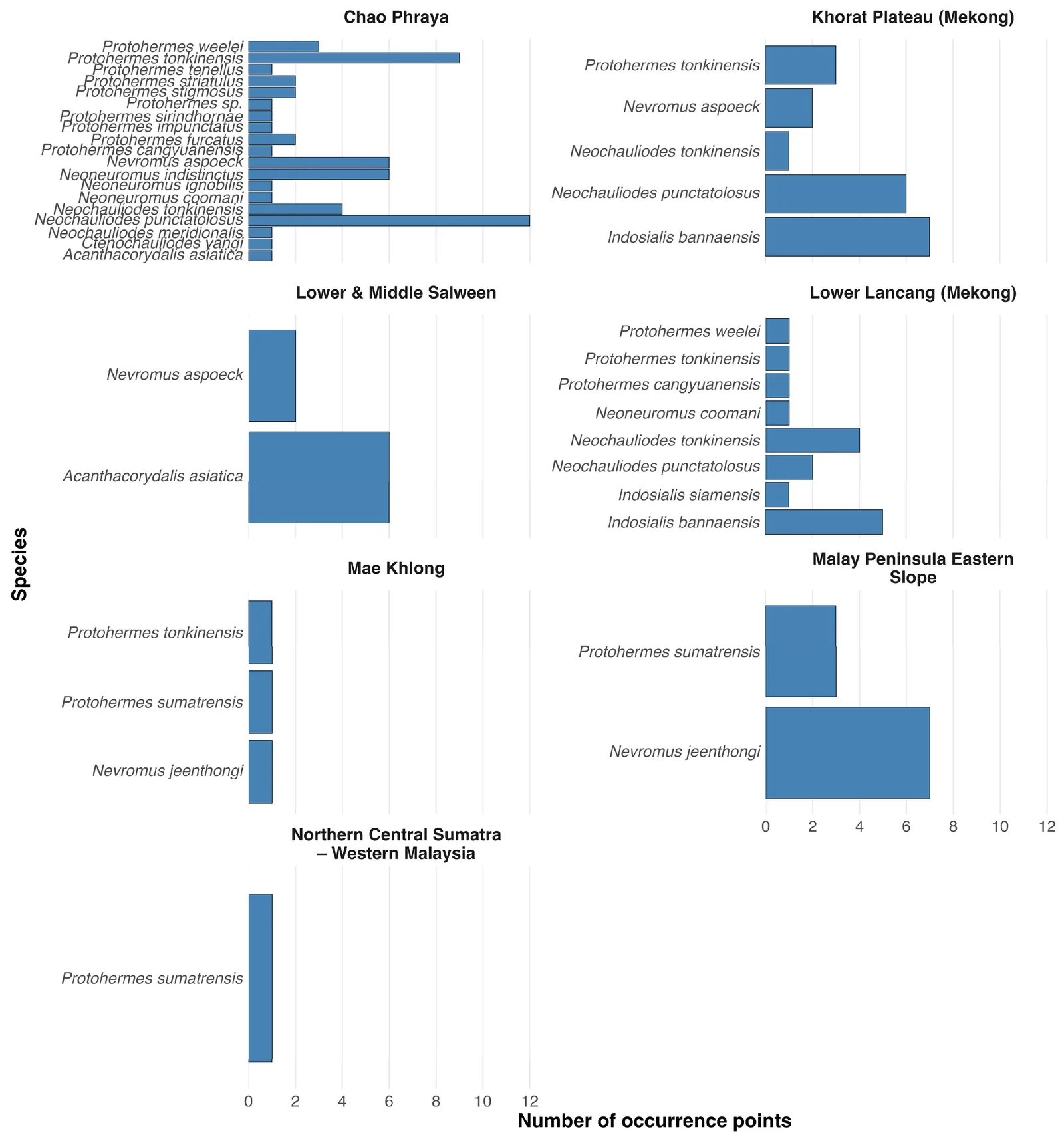
Distribution of 116 megalopteran occurrence records across seven freshwater ecoregions in Thailand. The number of occurrence points for each species within each ecoregion is indicated.

**Figure 4:**
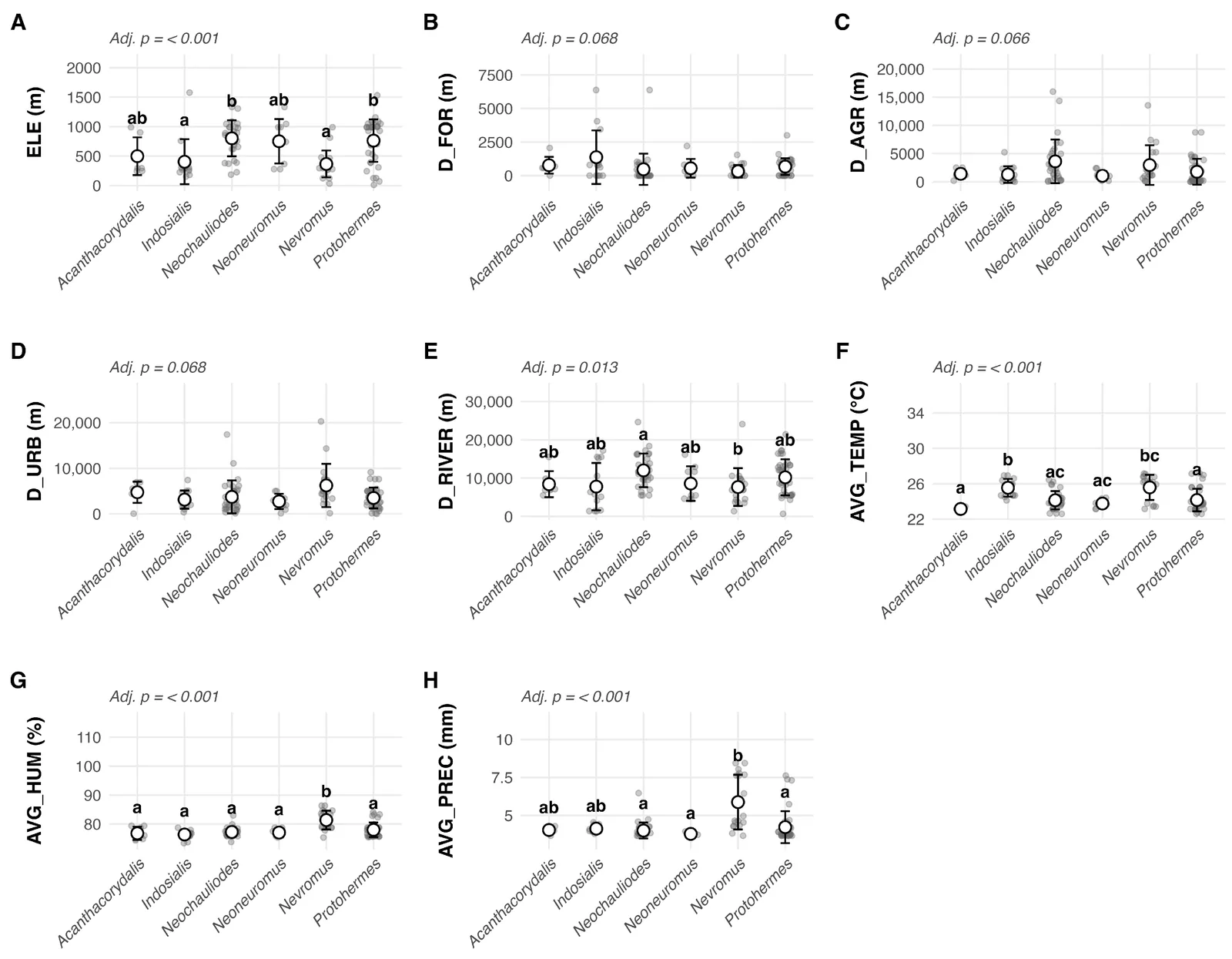
Mean (±SD) values of eight landscape and climatic variables across six megalopteran genera: (**A**) elevation (ELE), (**B**) distance to the nearest forest edge (D_FOR), (**C**) agricultural area (D_AGR), (**D**) urban area (D_URB), (**E**) distance to river or canal (D_RIVER), (**F**) annual mean temperature (AVG_TEMP), (**G**) annual mean humidity (AVG_HUM), and (**H**) annual mean precipitation (AVG_PREC). Significance letters from Dunn’s post-hoc tests are shown above genus means only when the corresponding adjusted Kruskal–Wallis test was significant, and adjusted *p*-values are reported within each panel.

**Figure 5:**
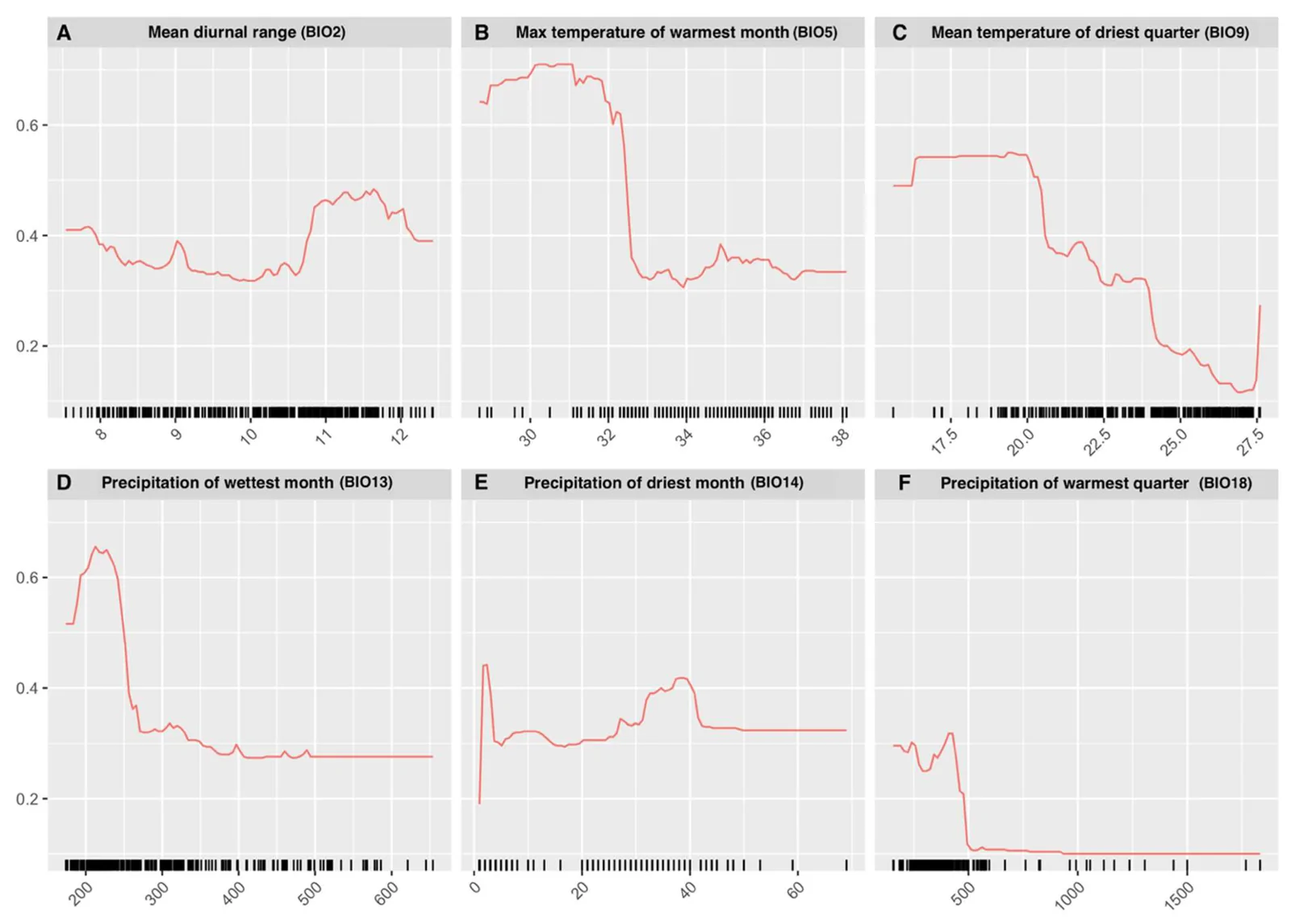
Response curves showing the predicted probabilities of occurrence for the overall megalopteran species in relation to six key bioclimatic variables: mean diurnal range (BIO2; (**A**)), max temperature of warmest month (BIO5; (**B**)), mean temperature of driest quarter (BIO9; (**C**)), precipitation of wettest month (BIO13; (**D**)), precipitation of driest month (BIO14; (**E**)), and precipitation of warmest quarter (BIO18; (**F**)).

**Figure 6:**
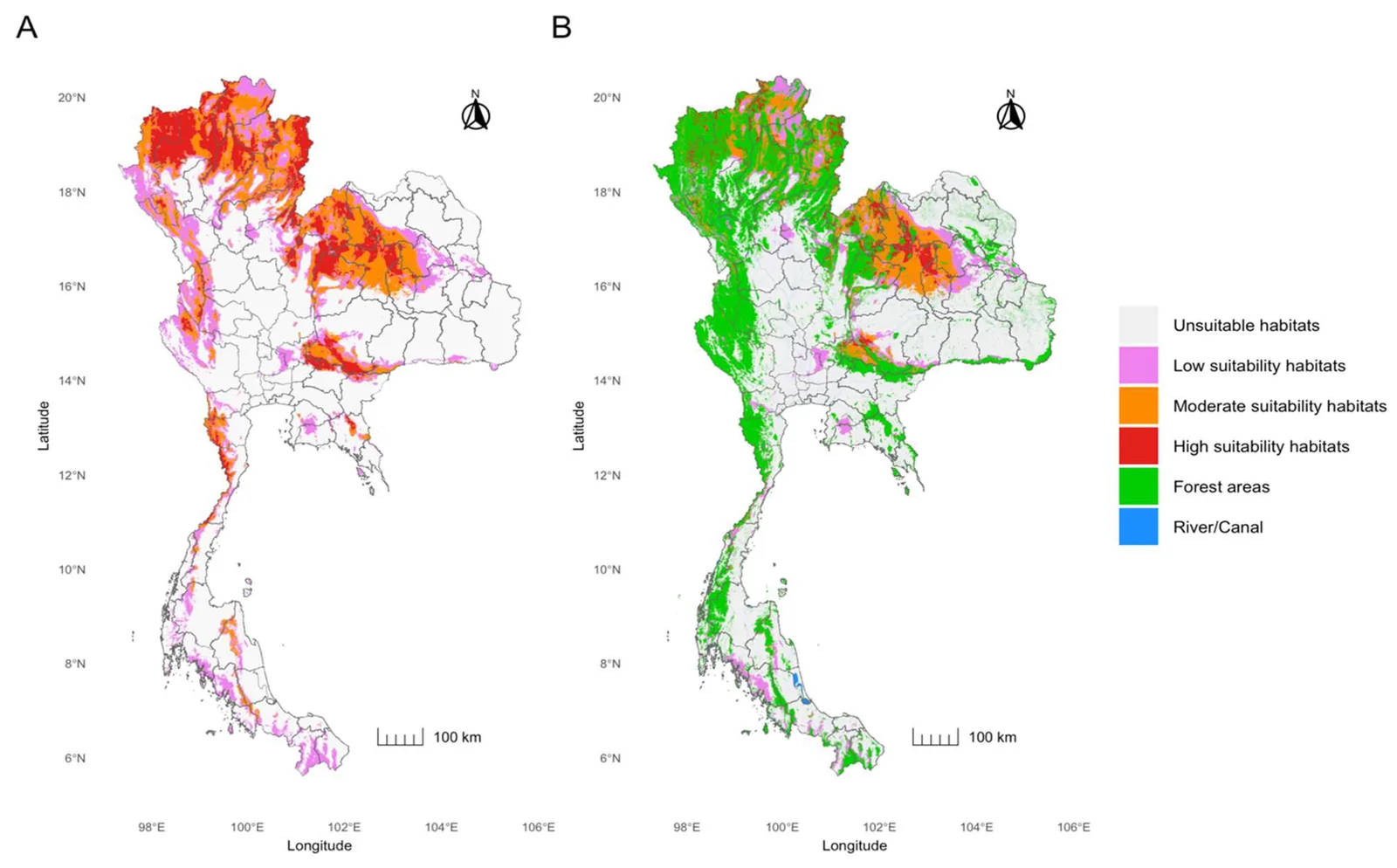
Habitat suitability maps for the overall megalopteran community in Thailand. The predicted habitat suitability was classified into four categories: unsuitable (<0.25), low (0.25 ≤ probability < 0.50), moderate (0.50 ≤ probability < 0.75), and high suitability (≥0.75) (**A**). Forested areas and river/canal networks were overlaid on the habitat suitability map to highlight priority areas for landscape management and conservation of the Thai megalopteran community (**B**).

**Figure 7:**
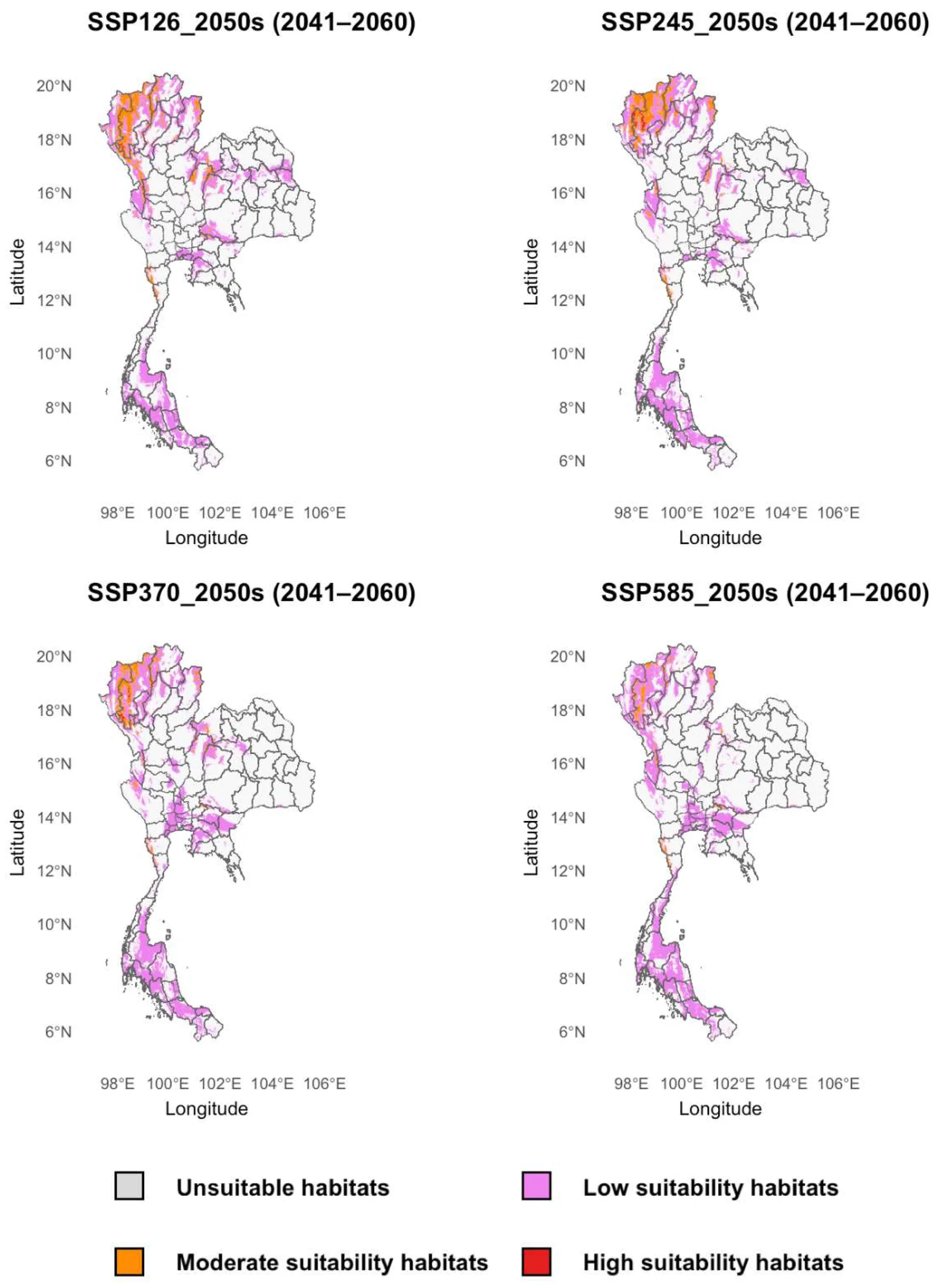
Predicted habitat suitability maps for the overall megalopteran community in Thailand under four future climate scenarios, representing Shared Socioeconomic Pathways (SSPs: SSP126, SSP245, SSP370, and SSP585) for the 2050s (2041–2060). Habitat suitability was classified into four categories: unsuitable (<0.25), low (0.25 ≤ probability < 0.50), moderate (0.50 ≤ probability < 0.75), and high suitability (≥0.75).

**Figure 8:**
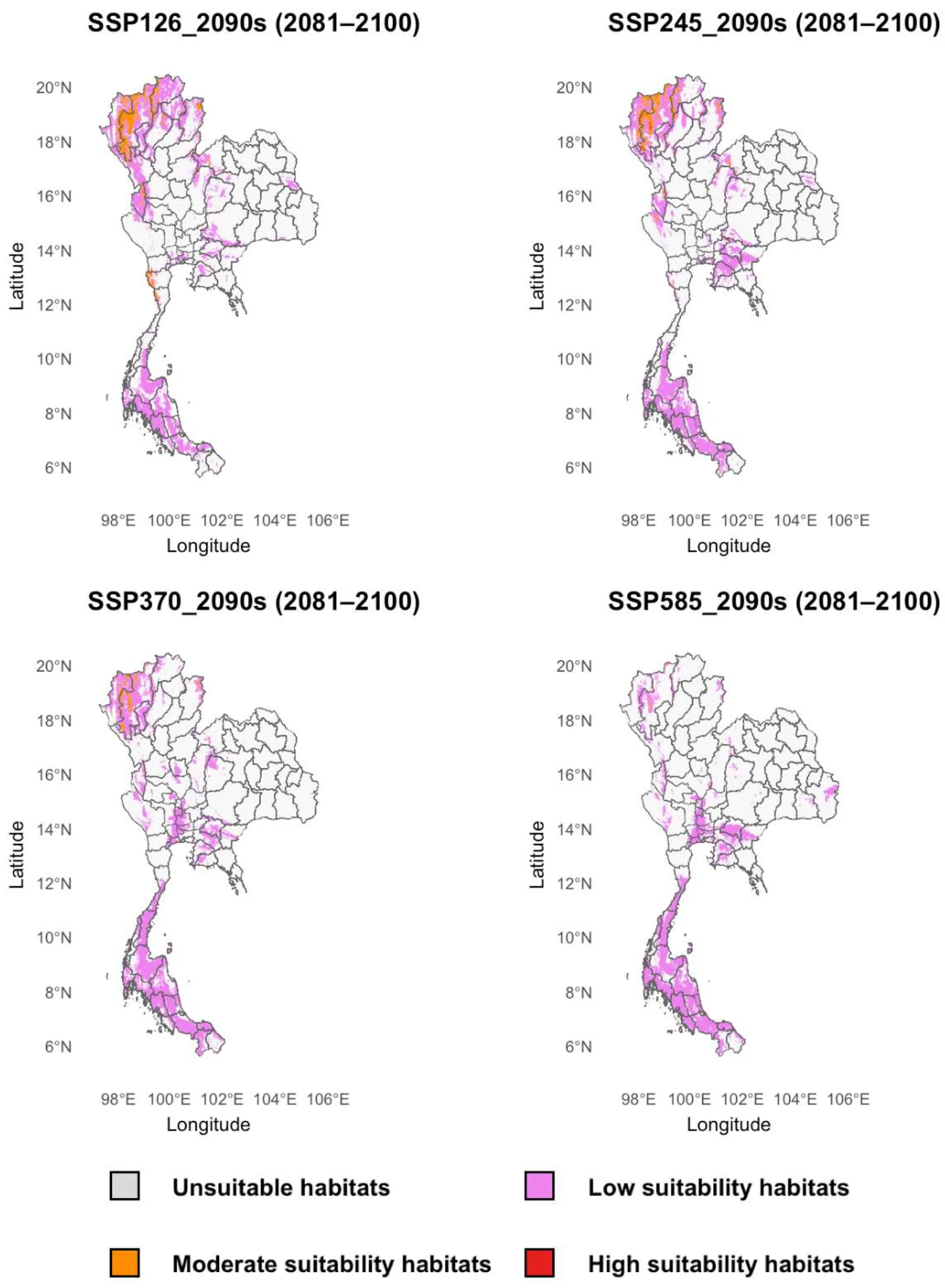
Predicted habitat suitability maps for the overall megalopteran community in Thailand under four future climate scenarios, representing Shared Socioeconomic Pathways (SSPs: SSP126, SSP245, SSP370, and SSP585) for the 2090s (2081–2100). Habitat suitability was classified into four categories: unsuitable (<0.25), low (0.25 ≤ probability < 0.50), moderate (0.50 ≤ probability < 0.75), and high suitability (≥0.75).

**Table 1:** Summary of megalopteran occurrence data, including family-, genus-, and species-level lists, and the total number of presence records for each genus and species. Occurrence records were compiled from three sources: (1) GBIF, (2) published literature, and (3) field surveys conducted in this study.

Family	Genus	No. of Occurrences	Species	No. of Occurrences	Source
Corydalidae	*Acanthacorydalis*	7	*Acanthacorydalis asiatica* (Wood-Mason, 1884)	7	(1), (2), (3)
	*Ctenochauliodes*	1	*Ctenochauliodes yangi* Liu & Yang, 2006	1	(2)
	*Neochauliodes*	31	*Neochauliodes meridionalis* van der Weele, 1909	1	(2)
			*Neochauliodes punctatolosus* Liu & Yang, 2006	21	(1), (2), (3)
			*Neochauliodes tonkinensis* (van der Weele, 1907)	9	(1), (2), (3)
	*Neoneuromus*	9	*Neoneuromus coomani* Lestage, 1927	2	(2)
			*Neoneuromus ignobilis* Navás, 1932	1	(2)
			*Neoneuromus indistinctus* Liu, Hayashi & Yang, 2018	6	(1), (2), (3)
	*Nevromus*	18	*Nevromus aspoeck* Liu, Hayashi & Yang, 2012	10	(1), (2), (3)
			*Nevromus jeenthongi* Piraonapicha, Jaitrong, Liu & Sangpradub, 2021	8	(1), (2), (3)
	*Protohermes*	37	*Protohermes* sp.	1	(3)
			*Protohermes cangyuanensis* Yang & Yang, 1988	2	(2)
			*Protohermes furcatus* Liu, Hayashi & Yang, 2008	3	(2)
			*Protohermes impunctatus* Liu, Hayashi & Yang, 2008	1	(2)
			*Protohermes sirindhornae* Piraonapicha, Chomphuphuang & Liu, 2025	1	(2)
			*Protohermes stigmosus* Liu, Hayashi & Yang, 2007	2	(2), (3)
			*Protohermes striatulus* Navás, 1926	2	(2), (3)
			*Protohermes sumatrensis* (van der Weele, 1909)	5	(1), (2), (3)
			*Protohermes tenellus* Liu, Hayashi & Yang, 2007	1	(2)
			*Protohermes tonkinensis* (van der Weele, 1909)	14	(1), (2), (3)
			*Protohermes weelei* Navás, 1925	5	(2), (3)
Sialidae	*Indosialis*	13	*Indosialis bannaensis* Liu, Yang & Hayashi, 2006	12	(2), (3)
			*Indosialis siamensis* Piraonapicha, Sangpradub, Jaitrong & Liu, 2020	1	(2)

**Table 2:** Potential suitable habitat area (km^2^) of megalopteran communities under current conditions and future climate change scenarios.

Scenario	Period	SSP	Low Suitability	Moderate Suitability	High Suitability	Total Area
Current	1970–2000	–	79,076.1 (15.4%)	73,530.6 (14.3%)	56,563.8 (11.0%)	209,170.5 (40.7%)
Future	2041–2060	SSP126	122,877.0 (23.9%)	30,567.3 (6.0%)	1270.1 (0.3%)	154,714.4 (30.1%)
Future	2081–2100	SSP126	115,992.7 (22.6%)	20,290.2 (4.0%)	174.1 (0.0%)	136,457.0 (26.6%)
Future	2041–2060	SSP245	115,105.8 (22.4%)	25,914.8 (5.0%)	2771.0 (0.5%)	143,791.6 (28.0%)
Future	2081–2100	SSP245	115,755.3 (22.5%)	15,543.0 (3.0%)	640.1 (0.1%)	131,938.4 (25.7%)
Future	2041–2060	SSP370	128,519.4 (25.0%)	20,332.7 (4.0%)	1036.8 (0.2%)	149,888.9 (29.2%)
Future	2081–2100	SSP370	117,208.3 (22.8%)	6364.6 (1.2%)	30.0 (0.0%)	123,602.9 (24.1%)
Future	2041–2060	SSP585	127,918.6 (24.9%)	11,335.1 (2.2%)	339.1 (0.1%)	139,592.8 (27.2%)
Future	2081–2100	SSP585	98,830.5 (19.2%)	583.7 (0.1%)	0.0 (0.0%)	99,414.2 (19.3%)

## Data Availability

The occurrence records, environmental predictor layers, and R code supporting the findings of this study are available from the corresponding author upon reasonable request.

## Supplementary Materials

The following supporting information can be downloaded at: https://www.mdpi.com/article/10.3390/insects17100986/s1, Table S1: Environmental variables used in the Kruskal–Wallis tests to characterize and compare environmental conditions associated with megalopteran genera; Table S2: Nineteen bioclimatic variables used for habitat suitability modelling; Table S3: Results of Kruskal–Wallis tests comparing environmental conditions associated with megalopteran genera, including distance to the nearest forest edge (D_FOR), agricultural area (D_AGR), urban area (D_URB), and river or canal (D_RIVER), as well as elevation (ELE), annual mean temperature (AVG_TEMP), annual mean humidity (AVG_HUM), and annual mean precipitation (AVG_PREC); Table S4: Results of Dunn’s post hoc tests comparing environmental conditions associated with megalopteran genera, including elevation (ELE), distance to the nearest river or canal (D_RIVER), annual mean temperature (AVG_TEMP), annual mean humidity (AVG_HUM), and annual mean precipitation (AVG_PREC); Table S5: Predicted potential habitat area (km^2^) of megalopteran communities under current conditions and future climate change scenarios; Figure S1: Correlation matrix of the six final bioclimatic variables used in the modeling process; Figure S2: Raster maps of six final bioclimatic variables used for the modelling process in the ‘biomod2’ package; Figure S3: Mean (±SD) evaluation scores for the validation dataset (AUC and TSS) for nine modeling algorithms used to predict the spatial distribution of the overall megalopteran community in Thailand; Figure S4: Averaged variable importance scores of six final bioclimatic variables influencing the spatial distribution of Megaloptera; Figure S5: Uncertainty map using a clamping mask in predicting habitat suitability of the overall megalopteran community across Thailand under current climate; Figure S6: Uncertainty map under future climate (SSP126; 2041–2060); Figure S7: Uncertainty map under future climate (SSP126; 2081–2100); Figure S8: Uncertainty map under future climate (SSP245; 2041–2060); Figure S9: Uncertainty map under future climate (SSP245; 2081–2100); Figure S10: Uncertainty map under future climate (SSP370; 2041–2060); Figure S11: Uncertainty map under future climate (SSP370; 2081–2100); Figure S12: Uncertainty map under future climate (SSP585; 2041–2060); Figure S13: Uncertainty map under future climate (SSP585; 2081–2100).

## Abbreviations

The following abbreviations are used in this manuscript:

D_FOR	Distance to nearest forest edge/patch
D_AGR	Distance to agricultural land
D_URB	Distance to urbanized land
D_RIVER	Distance to river/canal network
ELE	Elevation
AVG_TEMP	Average (annual mean) temperature
AVG_HUM	Average (annual mean) humidity
AVG_PREC	Average (annual mean) precipitation
BIO1–BIO19	The 19 standard bioclimatic variables
SSP	Shared socioeconomic pathway
SDM(s)	Species distribution model(s)
ANN	Artificial neural networks
CTA	Classification tree analysis
FDA	Flexible discriminant analysis
GAM	Generalized additive models
GBM	Generalized boosting method
GLM	Generalized linear models
MARS	Multivariate adaptive regression splines
MAXENT	Maximum entropy
RF	Random forest
SRE	Surface range envelope
PA(s)	Pseudo-absences
VIF	Variance Inflation Factor
AUC	Area under the (receiver operating characteristic) curve
TSS	True skill statistic
FDR	False discovery rate
GBIF	Global Biodiversity Information Facility
DEM	Digital elevation model (SRTM 90 m DEM)
SRTM	Shuttle radar topography mission
CIAT-CSI	International Center for Tropical Agriculture—Consortium for Spatial Information
FEOW	Freshwater ecoregions of the world
