# Supplementary material for: Habitat Suitability of Megalopteran Communities in Thailand: Conservation Implications Under Climate Change
Source: Insects. 2026 Sep 24;17(10):986. doi: 10.3390/insects17100986 (PMC13704958; doi:10.3390/insects17100986)
Supplement: Supplementary file 1 [file insects-17-00986-s001.zip › insects-4546798-supplementary.pdf]

Supplementary materials

Table S1. Environmental variables used in the Kruskal-Wallis tests to characterize and compare environmental conditions associated with Megaloptera genera.

Index	Abbreviation	Description for variable	Unit
Landscape variables	D_FOR	Distance to the nearest forest edge	m
	D_AGR	Distance to the nearest agricultural area	m
	D_URB	Distance to the nearest urban area	m
	D_RIVER	Distance to the nearest river/canal	m
Topographic variable	ELE	Elevation	m
Climatic variables	AVG_TEMP	Annual average temperature	℃
	AVG_HUM	Annual average humidity	%
	AVG_PREC	Annual average precipitation	mm

Table S2. Nineteen bioclimatic variables were used for habitat suitability modelling.

Index	Abbreviation	Description for variable	Unit
Bioclimatic variables	BIO1	Annual mean temperature	℃
	BIO2	Mean diurnal range	℃
	BIO3	Isothermality (BIO2/ BIO7) (*100)	℃
	BIO4	Temperature seasonality (standard deviation *100)	℃
	BIO5	Max temperature of warmest month	℃
	BIO6	Min temperature of coldest month	℃
	BIO7	Temperature annual range (BIO5- BIO6)	℃
	BIO8	Mean temperature of wettest quarter	℃
	BIO9	Mean temperature of driest quarter	℃
	BIO10	Mean temperature of warmest quarter	℃
	BIO11	Mean temperature of coldest quarter	℃
	BIO12	Annual precipitation	mm
	BIO13	Precipitation of wettest month	mm
	BIO14	Precipitation of driest month	mm

Index	Abbreviation	Description for variable	Unit
	BIO15	Precipitation seasonality (coefficient of variation)	1
	BIO16	Precipitation of wettest quarter	mm
	BIO17	Precipitation of driest quarter	mm
	BIO18	Precipitation of warmest quarter	mm
	BIO19	Precipitation of coldest quarter	mm

Table S3. Results of Kruskal-Wallis tests comparing environmental conditions associated with Megaloptera genera, including distance to the nearest forest edge (D_FOR), agricultural area (D_AGR), urban area (D_URB), and river or canal (D_RIVER), as well as elevation (ELE), annual mean temperature (AVG_TEMP), annual mean humidity (AVG_HUM), and annual mean precipitation (AVG_PREC).

Predictor	Chi_Sq	df	*p* value	Adjusted *p* value	Significance
ELE	28.433	5	< 0.001	< 0.001	***
D_FOR	10.278	5	0.068	0.068	ns
D_AGR	11.102	5	0.049	0.066	ns
D_URB	10.329	5	0.066	0.068	ns
D_RIVER	15.672	5	0.008	0.013	**
AVG_TEMP	34.895	5	< 0.001	< 0.001	***
AVG_HUM	27.343	5	< 0.001	< 0.001	***
AVG_PREC	33.888	5	< 0.001	< 0.001	***

Table S4. Results of Dunn's post hoc tests comparing environmental conditions associated with Megaloptera genera, including elevation (ELE), distance to the nearest river or canal (D_RIVER), annual mean temperature (AVG_TEMP), annual mean humidity (AVG_HUM), and annual mean precipitation (AVG_PREC).

**Predictor**	**Comparison**	**Z**	***p* value**	**Adjusted *p* value**
ELE	*Acanthacorydalis - Indosialis*	0.701	0.484	1.000
ELE	*Acanthacorydalis - Neochauliodes*	-1.998	0.046	0.686
ELE	*Indosialis - Neochauliodes*	-3.524	0.000	0.006
ELE	*Acanthacorydalis - Neoneuromus*	-1.396	0.163	1.000
ELE	*Indosialis - Neoneuromus*	-2.380	0.017	0.260
ELE	*Neochauliodes - Neoneuromus*	0.349	0.727	1.000
ELE	*Acanthacorydalis - Nevromus*	0.618	0.536	1.000
ELE	*Indosialis - Nevromus*	-0.146	0.884	1.000
ELE	*Neochauliodes - Nevromus*	3.751	< 0.001	0.003
ELE	*Neoneuromus - Nevromus*	2.398	0.016	0.247
ELE	*Acanthacorydalis - Protohermes*	-1.935	0.053	0.794
ELE	*Indosialis - Protohermes*	-3.493	< 0.001	0.007
ELE	*Neochauliodes - Protohermes*	0.158	0.875	1.000
ELE	*Neoneuromus - Protohermes*	-0.253	0.800	1.000
ELE	*Nevromus - Protohermes*	-3.734	< 0.001	0.003
D_RIVER	*Acanthacorydalis - Indosialis*	0.380	0.704	1.000
D_RIVER	*Acanthacorydalis - Neochauliodes*	-1.646	0.100	1.000
D_RIVER	*Indosialis - Neochauliodes*	-2.624	0.009	0.130
D_RIVER	*Acanthacorydalis - Neoneuromus*	0.206	0.837	1.000
D_RIVER	*Indosialis - Neoneuromus*	-0.172	0.863	1.000
D_RIVER	*Neochauliodes - Neoneuromus*	2.093	0.036	0.545
D_RIVER	*Acanthacorydalis - Nevromus*	0.693	0.488	1.000
D_RIVER	*Indosialis - Nevromus*	0.359	0.720	1.000
D_RIVER	*Neochauliodes - Nevromus*	3.366	0.001	0.011

Predictor	Comparison	Z	*p* value	Adjusted *p* value
D_RIVER	*Neoneuromus - Nevromus*	0.503	0.615	1.000
D_RIVER	*Acanthacorydalis - Protohermes*	-0.811	0.417	1.000
D_RIVER	*Indosialis - Protohermes*	-1.590	0.112	1.000
D_RIVER	*Neochauliodes - Protohermes*	1.456	0.145	1.000
D_RIVER	*Neoneuromus - Protohermes*	-1.178	0.239	1.000
D_RIVER	*Nevromus - Protohermes*	-2.238	0.025	0.378
AVG_TEMP	*Acanthacorydalis - Indosialis*	-4.657	< 0.001	< 0.001
AVG_TEMP	*Acanthacorydalis - Neochauliodes*	-2.672	0.008	0.113
AVG_TEMP	*Indosialis - Neochauliodes*	3.224	0.001	0.019
AVG_TEMP	*Acanthacorydalis - Neoneuromus*	-1.647	0.100	1.000
AVG_TEMP	*Indosialis - Neoneuromus*	3.121	0.002	0.027
AVG_TEMP	*Neochauliodes - Neoneuromus*	0.761	0.447	1.000
AVG_TEMP	*Acanthacorydalis - Nevromus*	-4.372	< 0.001	< 0.001
AVG_TEMP	*Indosialis - Nevromus*	0.648	0.517	1.000
AVG_TEMP	*Neochauliodes - Nevromus*	-2.799	0.005	0.077
AVG_TEMP	*Neoneuromus - Nevromus*	-2.738	0.006	0.093
AVG_TEMP	*Acanthacorydalis - Protohermes*	-2.506	0.012	0.183
AVG_TEMP	*Indosialis - Protohermes*	3.569	< 0.001	0.005
AVG_TEMP	*Neochauliodes - Protohermes*	0.350	0.726	1.000
AVG_TEMP	*Neoneuromus - Protohermes*	-0.546	0.585	1.000
AVG_TEMP	*Nevromus - Protohermes*	3.183	0.001	0.022
AVG_HUM	*Acanthacorydalis - Indosialis*	0.347	0.729	1.000
AVG_HUM	*Acanthacorydalis - Neochauliodes*	-0.420	0.674	1.000
AVG_HUM	*Indosialis - Neochauliodes*	-1.024	0.306	1.000
AVG_HUM	*Acanthacorydalis - Neoneuromus*	-0.160	0.873	1.000
AVG_HUM	*Indosialis - Neoneuromus*	-0.561	0.575	1.000
AVG_HUM	*Neochauliodes - Neoneuromus*	0.251	0.802	1.000
AVG_HUM	*Acanthacorydalis - Nevromus*	-3.241	0.001	0.018
AVG_HUM	*Indosialis - Nevromus*	-4.413	< 0.001	< 0.001

Predictor	Comparison	Z	*p* value	Adjusted *p* value
AVG_HUM	*Neochauliodes - Nevromus*	-4.278	< 0.001	< 0.001
AVG_HUM	*Neoneuromus - Nevromus*	-3.338	0.001	0.013
AVG_HUM	*Acanthacorydalis - Protohermes*	-0.827	0.408	1.000
AVG_HUM	*Indosialis - Protohermes*	-1.562	0.118	1.000
AVG_HUM	*Neochauliodes - Protohermes*	-0.678	0.498	1.000
AVG_HUM	*Neoneuromus - Protohermes*	-0.700	0.484	1.000
AVG_HUM	*Nevromus - Protohermes*	3.837	< 0.001	0.002
AVG_PREC	*Acanthacorydalis - Indosialis*	-0.437	0.662	1.000
AVG_PREC	*Acanthacorydalis - Neochauliodes*	1.122	0.262	1.000
AVG_PREC	*Indosialis - Neochauliodes*	2.042	0.041	0.618
AVG_PREC	*Acanthacorydalis - Neoneuromus*	1.794	0.073	1.000
AVG_PREC	*Indosialis - Neoneuromus*	2.558	0.011	0.158
AVG_PREC	*Neochauliodes - Neoneuromus*	1.148	0.251	1.000
AVG_PREC	*Acanthacorydalis - Nevromus*	-1.872	0.061	0.918
AVG_PREC	*Indosialis - Nevromus*	-1.727	0.084	1.000
AVG_PREC	*Neochauliodes - Nevromus*	-4.398	< 0.001	< 0.001
AVG_PREC	*Neoneuromus - Nevromus*	-4.257	< 0.001	< 0.001
AVG_PREC	*Acanthacorydalis - Protohermes*	1.433	0.152	1.000
AVG_PREC	*Indosialis - Protohermes*	2.469	0.014	0.204
AVG_PREC	*Neochauliodes - Protohermes*	0.498	0.619	1.000
AVG_PREC	*Neoneuromus - Protohermes*	-0.844	0.399	1.000
AVG_PREC	*Nevromus - Protohermes*	4.957	< 0.001	< 0.001

Table S5. Predicted potential habitat area (km²) of Megaloptera communities under current conditions and future climate change scenarios.

Scenario	Period	SSP	Area_Type	Category	Area (km^2)	% Area
Current	1970–2000	Baseline	Total (All Habitats)	Unsuitable	307843.3	59.9
Current	1970–2000	Baseline	Total (All Habitats)	Low suitability	79076.1	15.4
Current	1970–2000	Baseline	Total (All Habitats)	Moderate suitability	73530.6	14.3
Current	1970–2000	Baseline	Total (All Habitats)	High suitability	56563.8	11.0
Current	1970–2000	Baseline	Anthropogenic (No Forest)	Unsuitable	258866.9	50.4
Current	1970–2000	Baseline	Anthropogenic (No Forest)	Low suitability	39148.3	7.6
Current	1970–2000	Baseline	Anthropogenic (No Forest)	Moderate suitability	35933.1	7.0
Current	1970–2000	Baseline	Anthropogenic (No Forest)	High suitability	14978.2	2.9
Future	2050s (2041–2060)	SSP126	Total (All Habitats)	Unsuitable	362299.3	70.5
Future	2050s (2041–2060)	SSP126	Total (All Habitats)	Low suitability	122877.0	23.9
Future	2050s (2041–2060)	SSP126	Total (All Habitats)	Moderate suitability	30567.3	5.9
Future	2050s (2041–2060)	SSP126	Total (All Habitats)	High suitability	1270.1	0.2
Future	2090s (2081–2100)	SSP126	Total (All Habitats)	Unsuitable	380556.8	74.0
Future	2090s (2081–2100)	SSP126	Total (All Habitats)	Low suitability	115992.7	22.6
Future	2090s (2081–2100)	SSP126	Total (All Habitats)	Moderate suitability	20290.2	3.9
Future	2090s (2081–2100)	SSP126	Total (All Habitats)	High suitability	174.1	0.0
Future	2050s (2041–2060)	SSP245	Total (All Habitats)	Unsuitable	373222.2	72.6
Future	2050s (2041–2060)	SSP245	Total (All Habitats)	Low suitability	115105.8	22.4
Future	2050s (2041–2060)	SSP245	Total (All Habitats)	Moderate suitability	25914.8	5.0
Future	2050s (2041–2060)	SSP245	Total (All Habitats)	High suitability	2771.0	0.5
Future	2090s (2081–2100)	SSP245	Total (All Habitats)	Unsuitable	385075.2	74.9
Future	2090s (2081–2100)	SSP245	Total (All Habitats)	Low suitability	115755.3	22.5
Future	2090s (2081–2100)	SSP245	Total (All Habitats)	Moderate suitability	15543.0	3.0
Future	2090s (2081–2100)	SSP245	Total (All Habitats)	High suitability	640.1	0.1

Future	2050s (2041–2060)	SSP370	Total (All Habitats)	Unsuitable	367124.8	71.4
Future	2050s (2041–2060)	SSP370	Total (All Habitats)	Low suitability	128519.4	25.0
Future	2050s (2041–2060)	SSP370	Total (All Habitats)	Moderate suitability	20332.7	4.0
Future	2050s (2041–2060)	SSP370	Total (All Habitats)	High suitability	1036.8	0.2
Future	2090s (2081–2100)	SSP370	Total (All Habitats)	Unsuitable	393410.8	76.5
Future	2090s (2081–2100)	SSP370	Total (All Habitats)	Low suitability	117208.3	22.8
Future	2090s (2081–2100)	SSP370	Total (All Habitats)	Moderate suitability	6364.6	1.2
Future	2090s (2081–2100)	SSP370	Total (All Habitats)	High suitability	30.0	0.0
Future	2050s (2041–2060)	SSP585	Total (All Habitats)	Unsuitable	377420.9	73.4
Future	2050s (2041–2060)	SSP585	Total (All Habitats)	Low suitability	127918.6	24.9
Future	2050s (2041–2060)	SSP585	Total (All Habitats)	Moderate suitability	11335.1	2.2
Future	2050s (2041–2060)	SSP585	Total (All Habitats)	High suitability	339.1	0.1
Future	2090s (2081–2100)	SSP585	Total (All Habitats)	Unsuitable	417599.5	81.3
Future	2090s (2081–2100)	SSP585	Total (All Habitats)	Low suitability	98830.5	19.2
Future	2090s (2081–2100)	SSP585	Total (All Habitats)	Moderate suitability	583.7	0.1
Future	2090s (2081–2100)	SSP585	Total (All Habitats)	High suitability	0.0	0.0

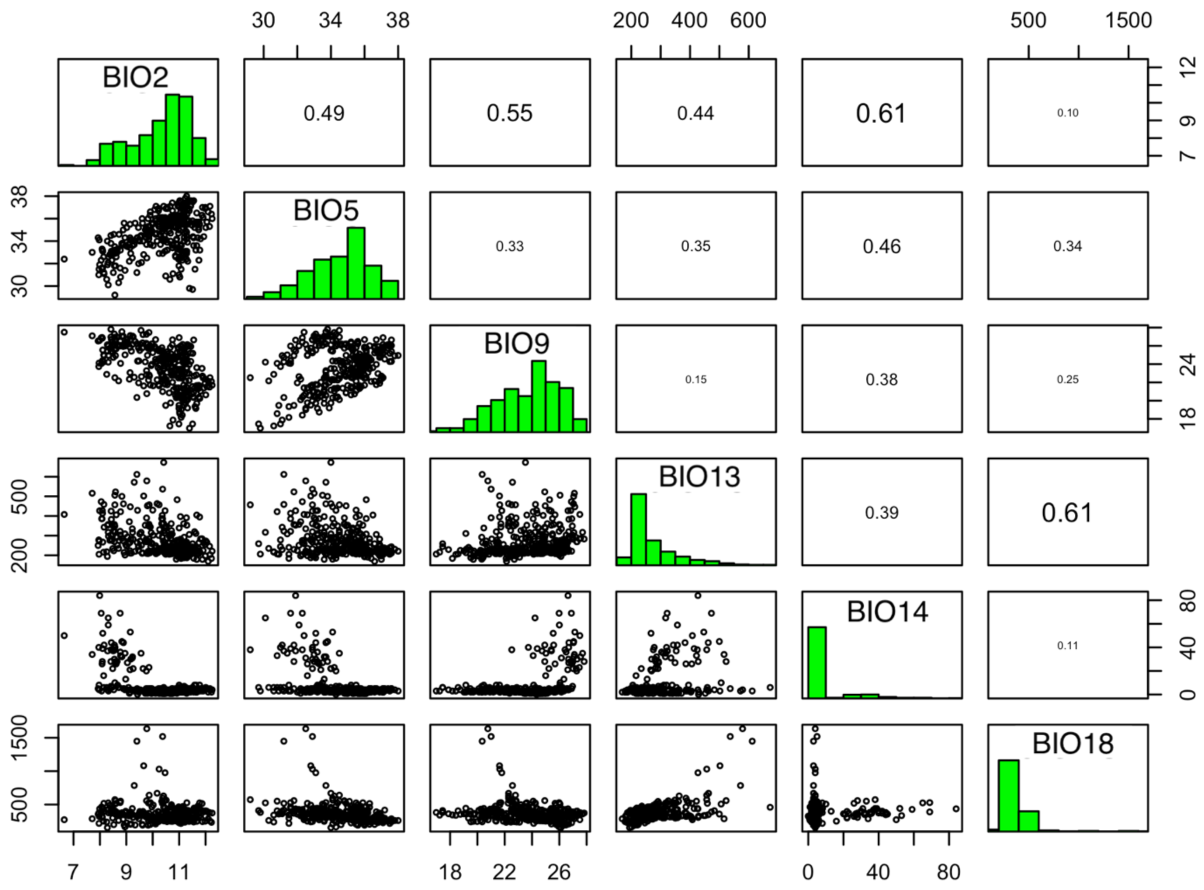

Figure S1. Correlation matrix of the six final bioclimatic variables used in the modeling process: mean diurnal range (BIO2), maximum temperature of the warmest month (BIO5), mean temperature of the driest quarter (BIO9), precipitation of the wettest month (BIO13), precipitation of the driest month (BIO14), and precipitation of the warmest quarter (BIO18).

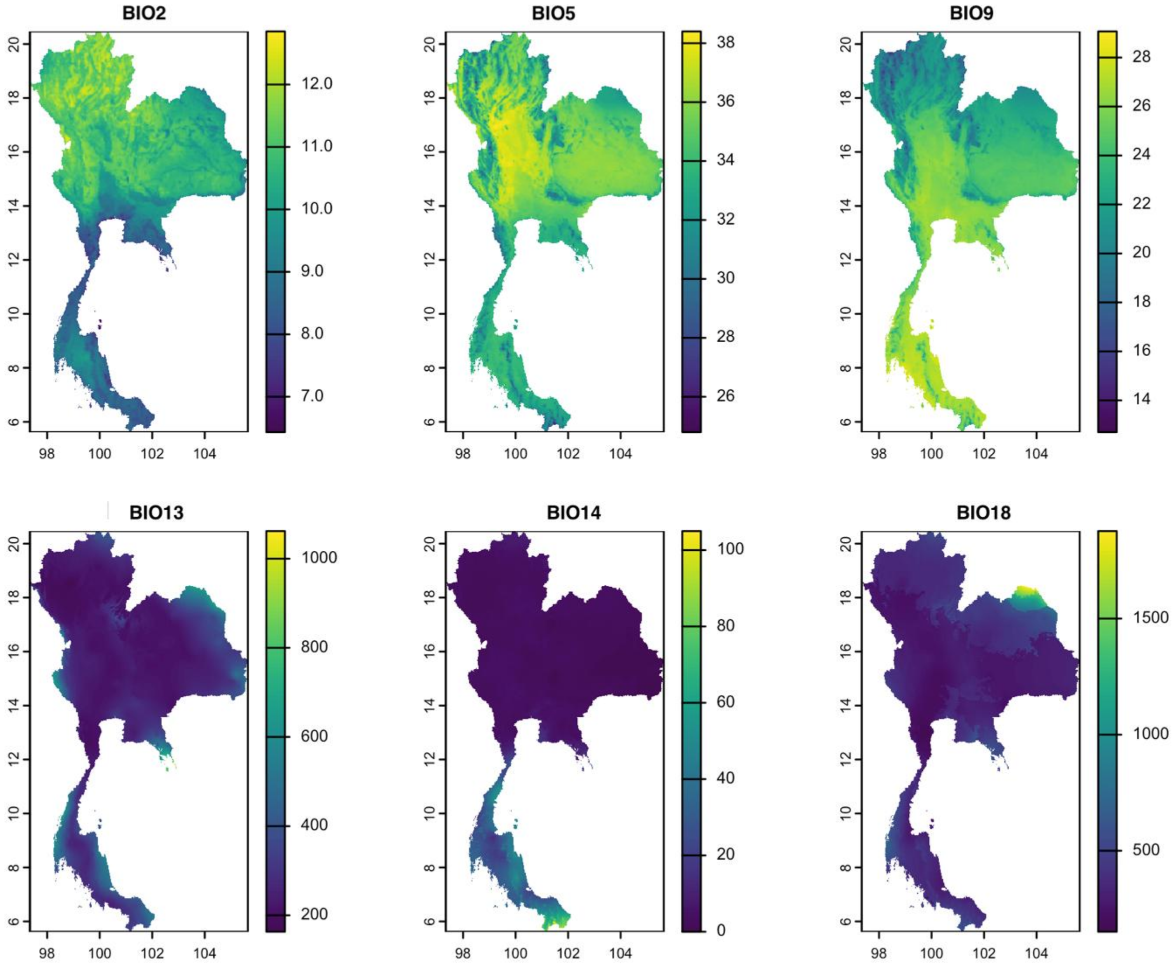

Figure S2. Raster maps of six final bioclimatic variables used for the modelling process in the 'biomod2' package: mean diurnal range (BIO2), maximum temperature of the warmest month (BIO5), mean temperature of the driest quarter (BIO9), precipitation of the wettest month (BIO13), precipitation of the driest month (BIO14), and precipitation of the warmest quarter (BIO18).

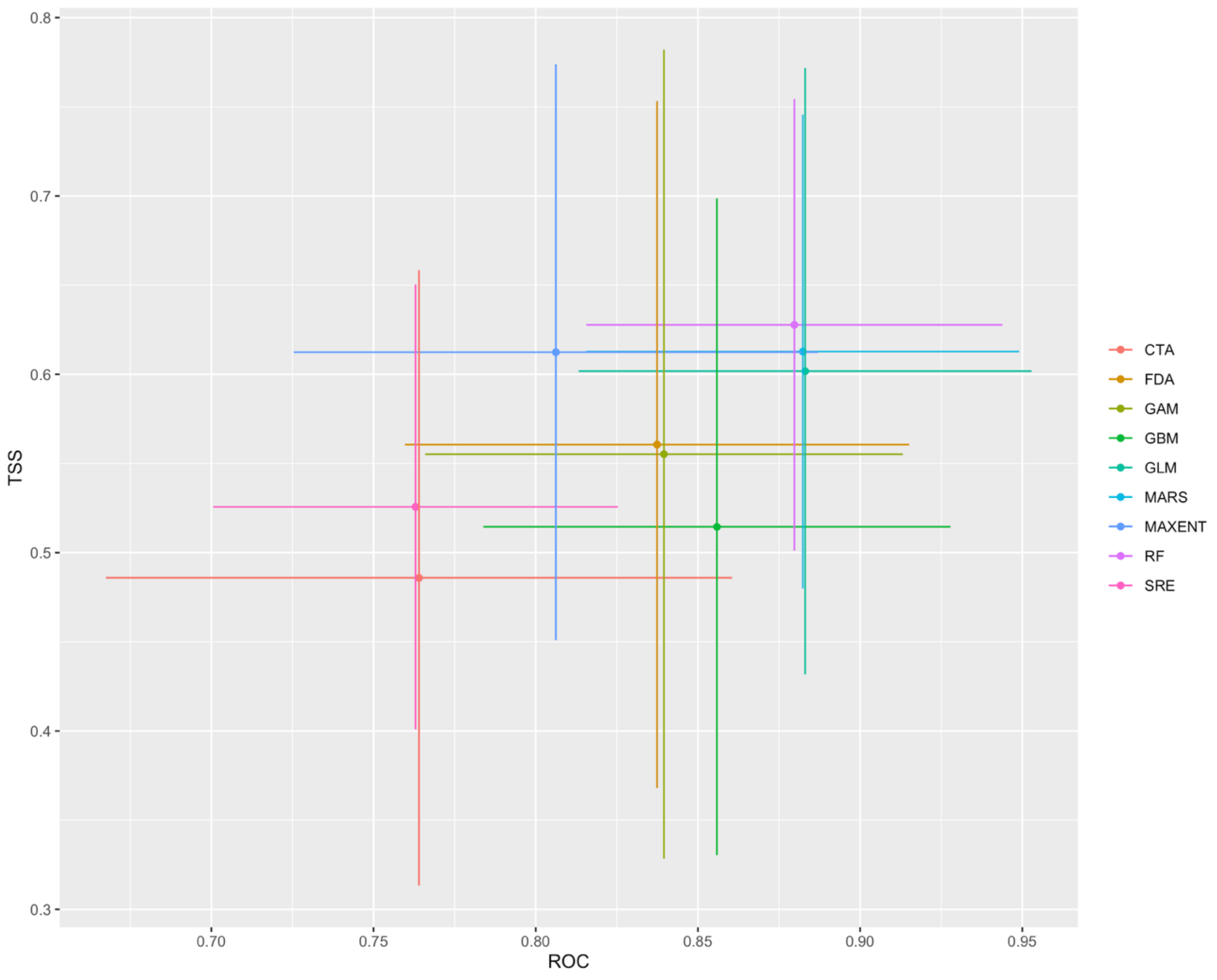

Figure S3. Mean (±SD) evaluation scores for the validation dataset, including the area under curve (AUC) values from the receiver operating characteristic (ROC) curve and true skill statistic (TSS), for nine modeling algorithms used to predict the spatial distribution of overall megalopteran community in Thailand. Ten predictive algorithms available in the biomod2 package were evaluated: artificial neural networks (ANN), classification tree analysis (CTA), flexible discriminant analysis (FDA), generalized additive models (GAM), generalized boosting method (GBM), generalized linear models (GLM), multivariate adaptive regression splines (MARS), maximum entropy (MAXNET), random forest (RF), and surface range envelope (SRE; also known as BIOCLIM). The RF algorithm was selected for habitat suitability map projection.

Artificial neural networks (ANN) failed during the modeling process and were excluded from the final evaluation.

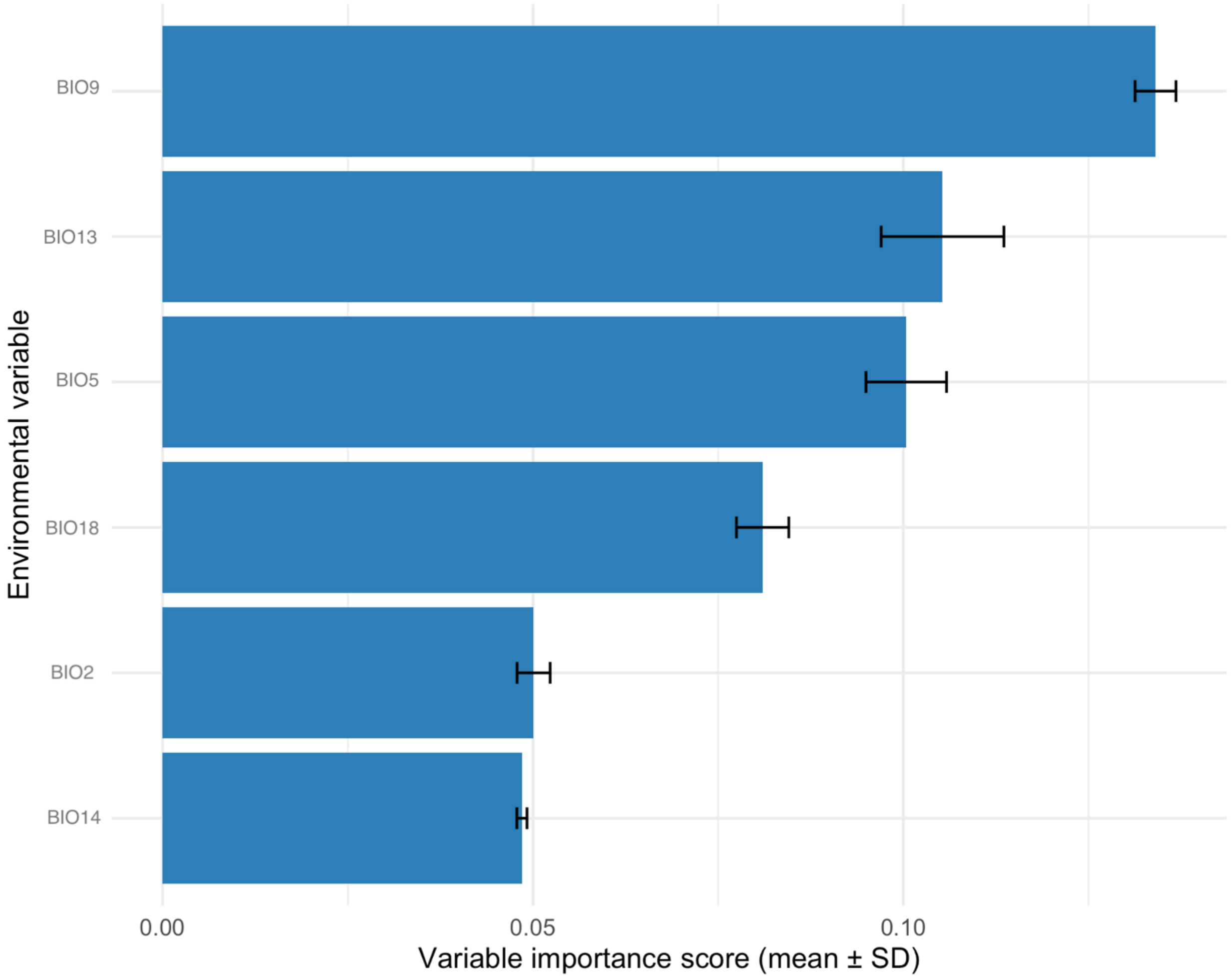

Figure S4. Averaged variable importance scores of six final bioclimatic variables influencing the spatial distribution of Megaloptera.

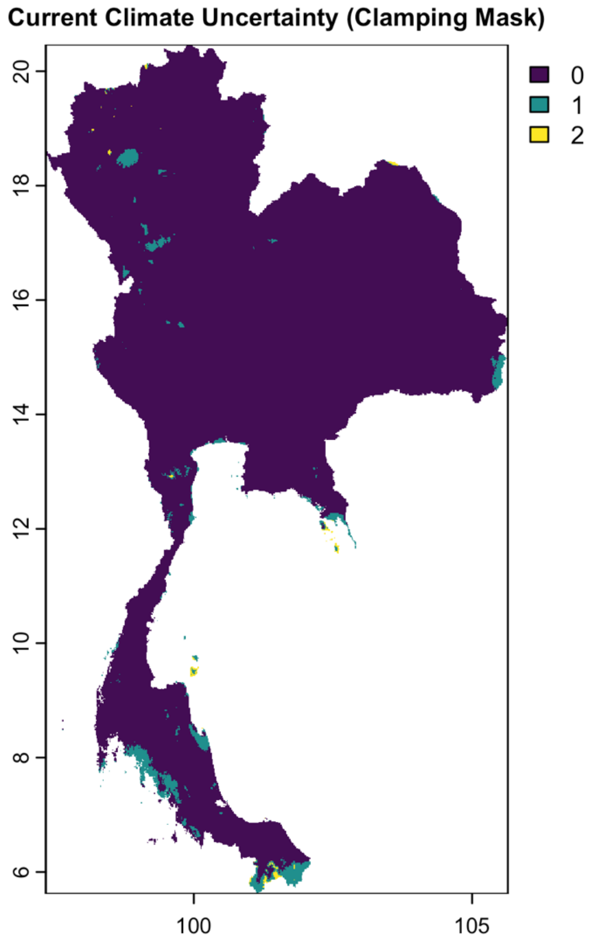

Figure S5. An uncertainty map using clamping mask in predicting the habitat suitability of overall megalopteran community across Thailand under current climate. The values will correspond to the number of variables in each pixel that are out of their calibration/validation range, identifying locations where predictions are uncertain.

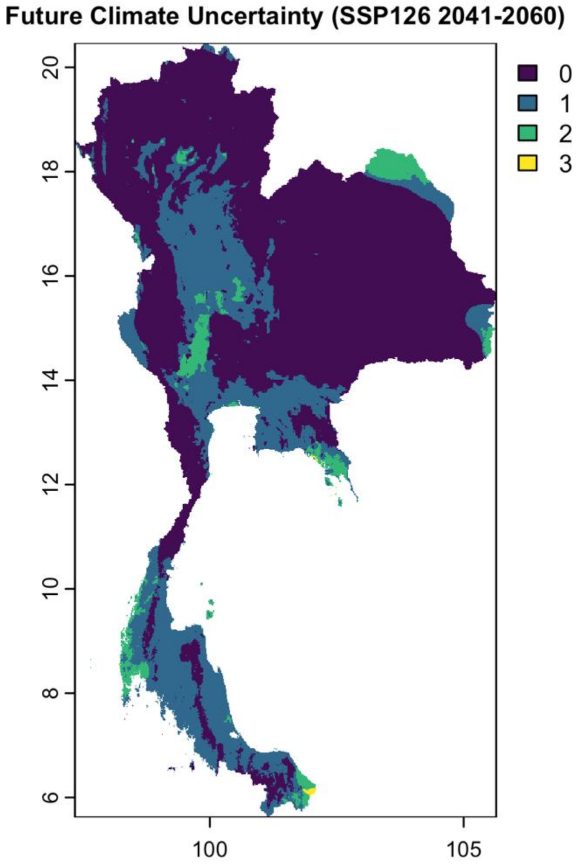

Figure S6. An uncertainty map using clamping mask in predicting the habitat suitability of overall megalopteran community across Thailand under future climate (SSP126; 2041–2060). The values will correspond to the number of variables in each pixel that are out of their calibration/validation range, identifying locations where predictions are uncertain.

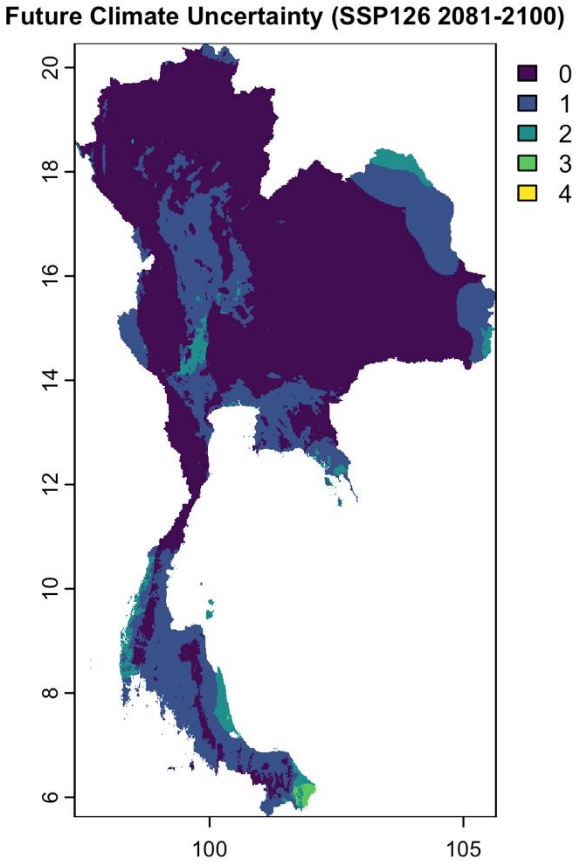

Figure S7. An uncertainty map using clamping mask in predicting the habitat suitability of overall megalopteran community across Thailand under future climate (SSP126; 2081–2100). The values will correspond to the number of variables in each pixel that are out of their calibration/validation range, identifying locations where predictions are uncertain.

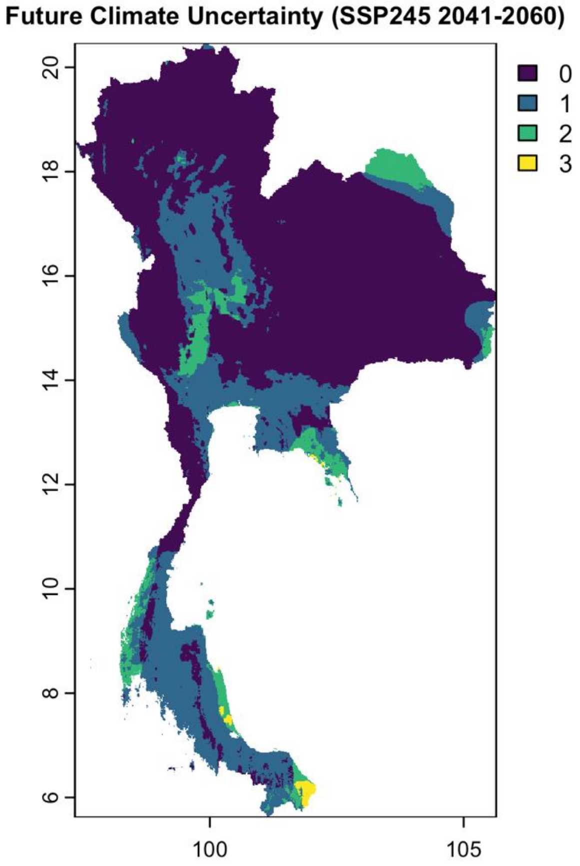

Figure S8. An uncertainty map using clamping mask in predicting the habitat suitability of overall megalopteran community across Thailand under future climate (SSP245; 2041–2060). The values will correspond to the number of variables in each pixel that are out of their calibration/validation range, identifying locations where predictions are uncertain.

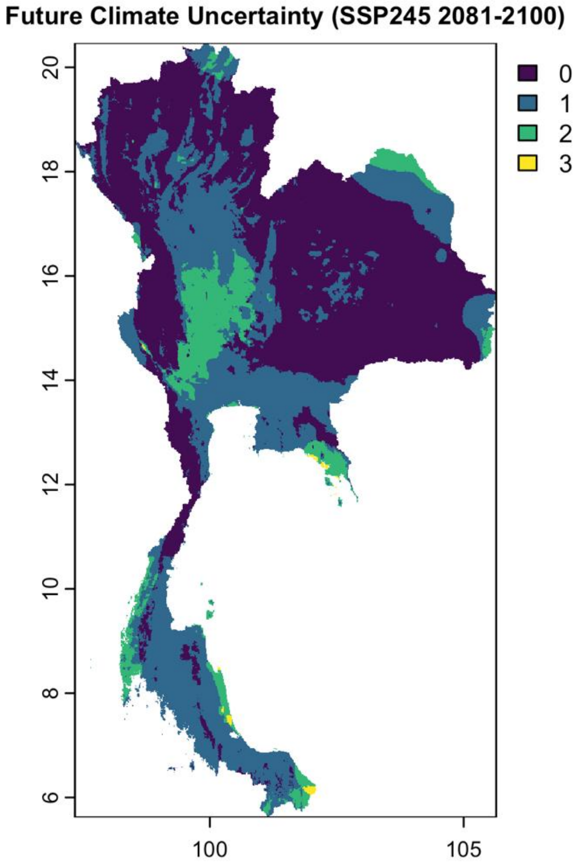

Figure S9. An uncertainty map using clamping mask in predicting the habitat suitability of overall megalopteran community across Thailand under future climate (SSP245; 2081–2100). The values will correspond to the number of variables in each pixel that are out of their calibration/validation range, identifying locations where predictions are uncertain.

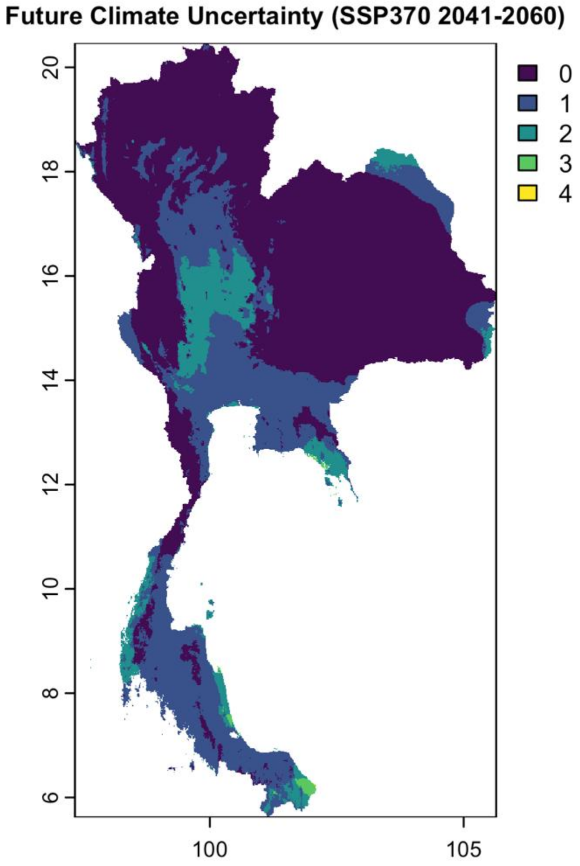

Figure S10. An uncertainty map using clamping mask in predicting the habitat suitability of overall megalopteran community across Thailand under future climate (SSP370; 2041–2060). The values will correspond to the number of variables in each pixel that are out of their calibration/validation range, identifying locations where predictions are uncertain.

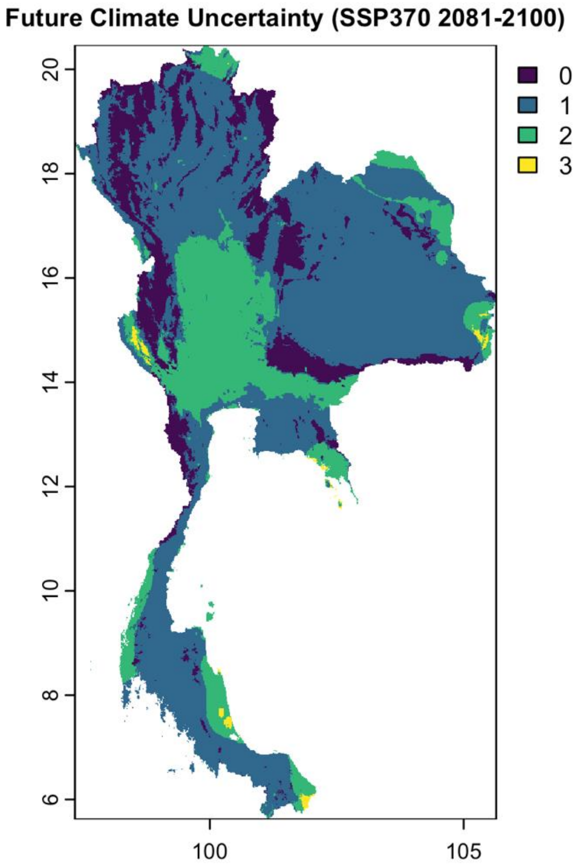

Figure S11. An uncertainty map using clamping mask in predicting the habitat suitability of overall megalopteran community across Thailand under future climate (SSP370; 2081–2100). The values will correspond to the number of variables in each pixel that are out of their calibration/validation range, identifying locations where predictions are uncertain.

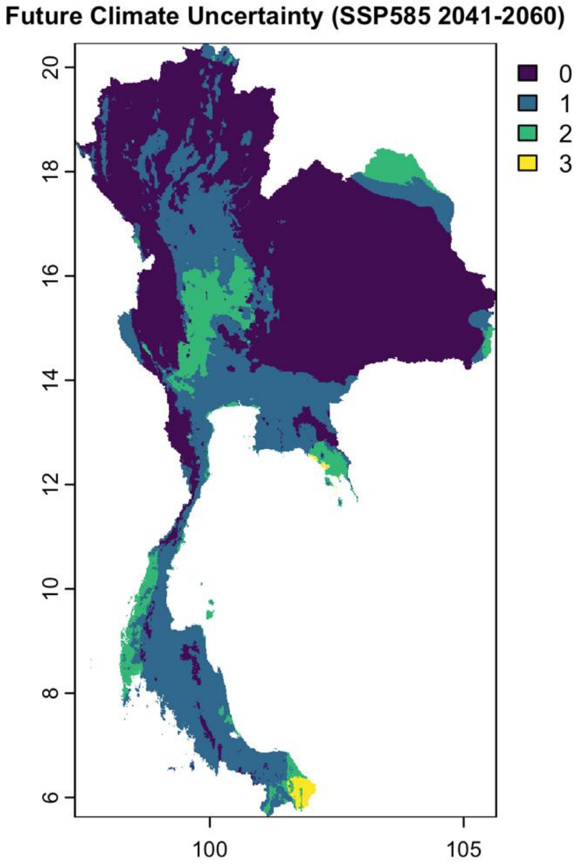

Figure S12. An uncertainty map using clamping mask in predicting the habitat suitability of overall megalopteran community across Thailand under future climate (SSP585; 2041–2060). The values will correspond to the number of variables in each pixel that are out of their calibration/validation range, identifying locations where predictions are uncertain.

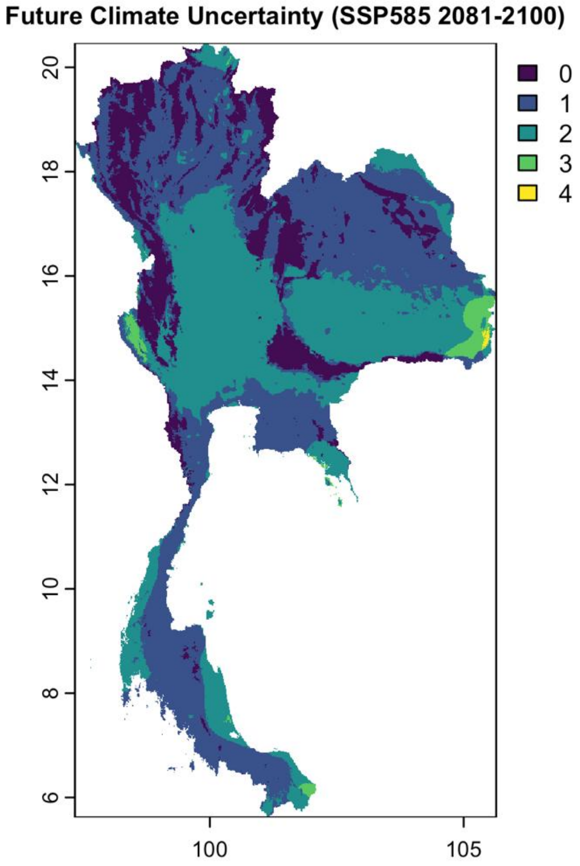

Figure S13. An uncertainty map using clamping mask in predicting the habitat suitability of overall megalopteran community across Thailand under future climate (SSP585; 2081–2100). The values will correspond to the number of variables in each pixel that are out of their calibration/validation range, identifying locations where predictions are uncertain.
